# A Coarse-Grained MARTINI Model for Mucins

**DOI:** 10.1021/acs.jctc.5c01655

**Published:** 2025-12-31

**Authors:** Thilakan Kanesalingam, Erik Weiand, Philippa M. Cann, Marc Masen, James P. Ewen

**Affiliations:** † Department of Mechanical Engineering, 4615 Imperial College London, South Kensington Campus, London SW7 2AZ, U.K.; ‡ Department of Mechanical Engineering, University of Bath, Claverton Down, Bath BA2 7AY, U.K.

## Abstract

Highly glycosylated proteins known as mucins are the principal components of mucus, the gel-like secretion that protects and lubricates many tissues in the human body. Molecular dynamics (MD) simulations are a useful tool to investigate the nanoscale structure and function of proteins; however, the high molecular weight of mucins makes them a challenging target for atomistic MD simulations. To enable long-time MD simulations of large mucins, we develop and validate new coarse-grained force field parameters within the MARTINI 3 framework for the glycosylated domains of salivary mucin, MUC5B. We use atomistic MD simulations of segments of the protein backbone connected to *O*-glycans with the CHARMM36m force field to parameterize the bonded parameters. The structural properties of MUC5B from the MD simulations with MARTINI 3, including the radius of gyration, end-to-end distance, and solvent accessible surface area, agree well with the atomistic simulations. Our MARTINI 3 parameters reproduce the bottlebrush structure of MUC5B observed in atomistic MD simulations and previous experiments. The power-law scaling of the radius of gyration with molecular weight is within the range observed in previous experiments of mucins. Accordingly, the MARTINI 3 parameters developed and validated in this study will facilitate accurate and efficient MD simulations of mucins and other glycoproteins for a variety of application areas including food science, drug delivery, and biomaterials.

## Graphical abstract

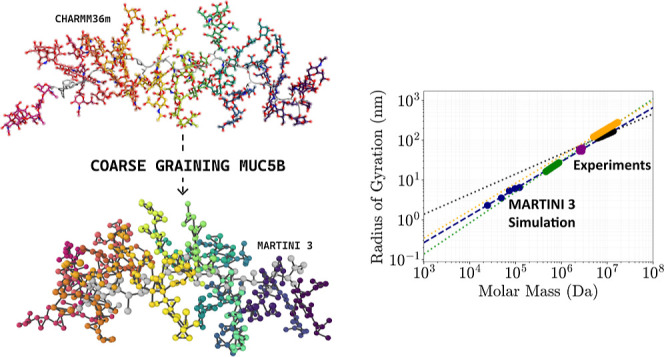

## Introduction

Mucus is a hydrogel that covers the mucosal surfaces in our body, including the mouth, intestines, stomach, eyes, and lungs.^1^ It performs several important functions, including acting as a barrier by lubricating surfaces, protecting cells, selectively allowing the passage of nutrients, and helping to remove pathogens and debris.^2^ Mucus is also home to our microbiome^3^ and acts as a reservoir of complex carbohydrate structures known as glycans, which play critical roles in controlling cell adhesion^4^ and signaling.^5^ In addition to their many functions in the human body,^1^ mucins are also emerging as a useful building block for biomaterials.^6^

Mucus is mostly composed of water (∼95%), but also comprises of salts, lipids, phospholipids, cholesterol, and several proteins.^7^ The primary components that drive the viscous, gel-like properties of mucus are large glycoproteins known as mucins.^7^ Different mucins are secreted by cells on the various mucosal surfaces in the body,^1^ for example, mucin MUC5B is the dominant mucin in the mouth and lungs,^8^ while mucin MUC5AC is most common in the stomach and eyes.^9^

Mucins are surfactant-like proteins that consist of both hydrophobic and hydrophilic domains.^7^ The main structural domains of the mucin, MUC5B, are illustrated in Figure 1A. Mucins contain an intrinsically disordered (IDP) backbone in the central domains of the protein, which mostly comprises of proline, threonine, and serine (PTS) amino acids.^10,11^ The PTS-rich domains are densely modified by negatively charged *O*-glycans, which makes these regions hydrophilic.^10^ These glycosylated domains form extended bottlebrush structures,^10^ which give rise to a range of properties of mucins, including their lubricating, antifouling, and water-retaining abilities.^12^ The central PTS-rich domains alternate with cysteine-rich domains that are hydrophobic^12^ and form compact β-sheet structures.^13^ The ends of the protein are made up of von Willebrand factor (vWF)-like N-terminal and C-terminal domains, which are also hydrophobic.^12^ These hydrophobic regions facilitate the formation of gel-like networks^14^ and promote surface adhesion.^15^

**Figure 1:**
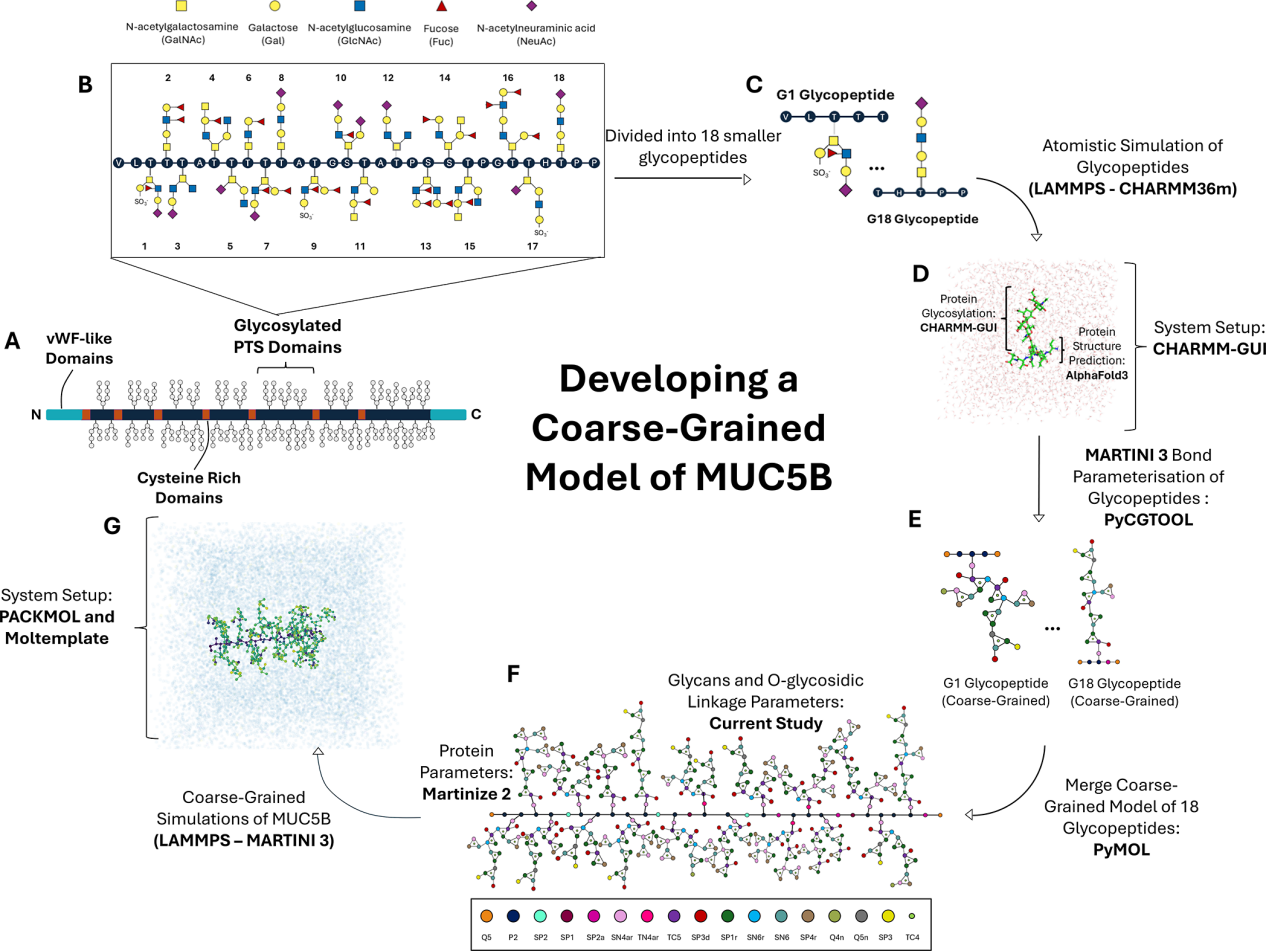
Schematic showing the workflow used in this study to obtain the MARTINI 3 ^24^ parameters for mucins. (A) Illustration of the protein structure of MUC5B adapted from Figure 1C of Kearns et al.^10^ The central PTS-rich domains (navy) are glycosylated with negatively charged *O*-glycans (gray). These PTS-rich domains are interspersed between cysteine-rich domains (orange), with the protein terminated by vWF-like domains (light blue). (B) Atomistic Mini2 structure of MUC5B adapted from Figure 3B­(i) of Kearns et al.^10^ drawn with symbol nomenclature for glycans (SNFG) representation and color scheme.^61^ A key of the sugars present in the glycans is provided. (C) SNFG representation^61^ of the G1 glycopeptide and G18 glycopeptide, two of 18 glycopeptides used for bonded parameter development. (D) PyMOL^62^ rendering of atomistic Molecular Dynamics (MD) simulation of G1 glycopeptide with CHARMM36m.^48^ (E) Coarse-grained (CG) MARTINI 3 mapping of G1 and G18 glycopeptide. (F) CG MARTINI 3 mapping of MUC5B glycopeptide. (G) OVITO^63^ rendering of CG MD simulation of MUC5B with MARTINI 3.^24^ Parts (A) and (B) are adapted from Kearns et al.^10^ (available under a CC-BY-NC-ND 4.0 License. Copyright 2024 F.L. Kearns, M.A. Rosenfeld and R.E. Amaro).

Over the last few decades, molecular dynamics (MD) simulations have become a valuable tool with which the structure and function of proteins are studied.^16^ Given the widespread importance of mucins, surprisingly few examples of MD simulations of them are available in the literature. This is likely because mucins are large proteins (2–50 MDa)^7^ with complex structures, and their slow dynamics makes MD simulations with atomistic force fields a considerable challenge.^10^ Even with access to exascale computers, MD simulations of large proteins with atomistic force fields are limited to microsecond time scales.^17^ Consequently, coarse-grained (CG) force fields, which group atoms into beads to reduce the number of interaction sites, have become a key strategy in protein modeling.^18^ Compared to atomistic force fields, CG force fields can reduce the computational cost of biomolecular simulations by an order of magnitude or more.^18^ Only a few CG MD simulations of mucins have been reported, including the study by Javitt et al.^19^ who investigated the assembly of intestinal mucin (MUC2) with vWF factor polymers and Ford et al.^20^ who explored the structure and rheological properties of MUC5B. Recently, Biswas and Levy^21^ developed a CG model of the disordered PTS-rich region of MUC2, MUC5AC, and MUC5B to study the effects of glycosylation and ionic conditions on the structural properties of mucins. To our knowledge, mucins have not yet been modeled with general-purpose CG force fields, such as MARTINI. Originally developed for lipid-based systems,^22^ the MARTINI model has been continually developed over the last two decades and can now be used to simulate a wide range of biological systems.^23^ Released in 2021,^24^ the MARTINI 3 model has already been extended to mucin-like systems, including intrinsically disordered proteins (backbone)^25,26^ and carbohydrates (glycans).^27,28^ While MARTINI parameters are available for *N*-glycans,^29,30^ bonded parameters have not yet been developed for the *O*-glycans found on MUC5B and other mucins. Accurate models of negatively charged glycans are important because they give rise to the bottlebrush structure of mucins, which is known to be critical to their adsorption and lubrication performance.^31^

In this study, we develop and validate MARTINI 3 parameters for mucins and showcase their transferability by performing MD simulations of the glycosylated domains of MUC5B. This is expected to facilitate CG MD simulations of mucins with MARTINI for a wide range of important application areas such as food science,^32^ drug delivery,^33^ and biomaterials.^34^ Our parametrization includes extension of MARTINI to *O*-glycans, which are important not only for mucins but also for lubricin,^35^ as well as serum and cell membrane glycoproteins.^36^

## Methodology

### Atomistic MD Simulations for Parameterization

We performed MD simulations of short MUC5B glycopeptides with an atomistic force field to develop MARTINI 3-bonded parameters. We utilized the Mini2 structure described by Kearns et al.^10^ with the protein backbone representing a 30-amino acid consensus sequence of the MUC5B PTS domain, as determined by Hughes et al.^11^ A schematic of the sequence is shown in Figure 1B. A larger version of Figure 1B is provided in the Supporting Information (Figure S1), with the glycosidic linkages labeled. We attached 18 *O*-glycans to the 30 amino acid peptide, as described by Kearns et al.,^10^ who derived the composition of the *O*-glycans using mass spectrometry data from Thomsson et al.^37^ in conjunction with the GlyConnect Expasy database.^38^ Although mucins also contain vWF-like C- and N-terminal and cysteine-rich domains, these only represent a small fraction of their total molecular mass.^7^ Thus, the focus of our parameterization is the heavily *O*-glycosylated PTS-rich domain of MUC5B, which accounts for over 80% of the total mass.^7^ To reduce the computational cost of the atomistic simulations for the parameterization, we split the MUC5B Mini2 structure^10^ into 18 smaller glycopeptides, each containing five amino acids and one *O*-glycan, as shown in Figure 1C. The initial structure of the glycopeptide backbones was predicted using AlphaFold 3.^39,40^ The protein backbones were glycosylated using the Glycan Reader & Modeler tool^41–43^ in CHARMM-GUI.^44,45^ The Glycan Reader & Modeler tool^41–45^ was subsequently used to assign the glycopeptides with the atomistic CHARMM36m force field.^46–48^ The resulting systems were solvated with TIP3P water molecules^49^ using CHARMM-GUI.^41–45^ Sodium counterions were added to neutralize the negatively charged glycans, with additional sodium and chloride ions added to give a background salt concentration of 150 mM NaCl. This concentration is commonly used as a physiological salt concentration in MARTINI 3 simulations^26,50^ and is within the range measured in human saliva 12–217 mM.^51^ Previous CG MD simulations showed that 20 mM NaCl concentrations lead to a reduction in the radius of gyration of mucin by around a third compared to the salt-free condition, with the value falling slightly when the salt concentration was further increased to 150 mM.^21^ The exact number of water molecules and Na^+^ and Cl^*–*^ ions for each glycopeptide system is further detailed in the Supporting Information (Table S1).

The resulting systems were used to run atomistic MD simulations using the Large-scale Atomic/Molecular Massively Parallel Simulator (LAMMPS) software.^52^ All MD simulations use the velocity-Verlet integration scheme.^53^ Each atomistic glycopeptide system underwent an energy minimization using the conjugate gradient algorithm. A short equilibration was then performed for 20 ps in the canonical (*NVT*) ensemble using the Nosé-Hoover thermostat^54,55^ set to a target temperature of 300 K. This was followed by a longer equilibration under the isothermal–isobaric (*NPT*) ensemble for a further 5 ns. The temperature and pressure during the *NPT* equilibration was controlled at 300 K and 1 atm using the Nosé-Hoover thermostat^54,55^ and barostat.^56^ Following equilibration, the production runs of each of the glycopeptides were carried out under the *NVT* ensemble at 300 K for a cumulative time of 500 ns. A time step, Δ*t*, of 2 fs was used throughout the *NPT* equilibration and production runs, which was enabled through constraining the bonds connected to hydrogen atoms with the SHAKE algorithm.^57^ The Lennard-Jones interactions were calculated using the CHARMM force switching function,^58^ with the van der Waals forces gradually switched off between 1.0 and 1.2 nm. The long-range Coulombic interactions were calculated using the particle–particle particle-mesh method (PPPM).^59,60^

### Coarse-Grained Mapping

In MARTINI 3, two to four heavy (non-hydrogen) atoms are combined into a single interaction center known as a CG bead.^24^ Different types of CG beads can represent two (tiny bead, T), three (small bead, S), or four (regular bead, R) heavy atoms.^24^ The mapping for the sugars mostly follows the bead types assigned in previous MARTINI 3 studies of monosaccharides^28^ and disaccharides.^28,64^ Previous MARTINI 3 parameters are available for the glycosidic linkages between monosaccharide units,^65^ but these are only compatible with an alternative carbohydrate mapping and parameterization^27^ to the one used here.^28^ The CG beads were placed at the center of geometry of its constituent atoms by the PyCGTOOL package.^66^ When grouping the atomic structures of the polysaccharides into beads for CG mapping, the glycosidic oxygen connecting the two sugars was assigned to the glycosyl acceptor sugar, in line with the studies by Grünewald et al.^28^ and Brandner et al.^64^ Furthermore, since the formation of a glycosidic bond involves the removal of a hydroxyl group from one sugar and a hydrogen from the other sugar, the mapping of the beads comprising the glycosidic bonds were accordingly adjusted from the standard mapping of monosaccharides.^28^ To our knowledge, the formation of a glycosidic bond between *N*-acetylhexosamines (i.e., *N*-acetylgalactosamine) and another monosaccharide has not previously been mapped with MARTINI 3. The group containing the anomeric carbon of *N*-acetylhexosamines, –C­(H) (OH)–CH–, is typically assigned an SN6 bead, as per the monosaccharide mapping by Grünewald et al.^28^ However, the removal of a hydroxyl group upon formation of a glycosidic bond leaves behind a –CH–CH- group as part of the five-membered carbon ring (colored in purple in Figure 2A). A TC5 bead was used to represent this moiety, with its polarity level of 5 being slightly higher than that typically assigned for carbons as part of aliphatic cyclic rings (polarity level of 3), reflecting the increased polarity of the two carbons substituted with a polar, electron-withdrawing amide (shown in red in Figure 2A) and glycosidic oxygen moiety (shown in pink in Figure 2A). Furthermore, while the protein–glycan linkage has previously been parameterized with the MARTINI 2 force field^67^ by Chakraborty et al.,^30^ to date we are unaware of parameterization of the *O*-glycosidic linkage of glycans to proteins using the MARTINI 3 force field. Drawing from the parameterization of similar chemical structures with the MARTINI 3 force field,^28,68^ we assigned SN4ar and TN4ar beads for the Threonine–glycan and Serine–glycan glycosidic linkage, respectively. We have also further extended the mapping of saccharide structures by including a Q4n bead to map the sulfate groups present on sulfated *O*-glycans, in line with previous mapping of the sulfate headgroup of dodecyl sulfate.^69^ The mapping of the protein backbone and the side chains of the unglycosylated amino acids followed standard MARTINI 3 mapping of amino acids. While the glycopeptides comprised largely of Threonine, Serine, and Proline residues in their backbone, whose side chains are nonionizable at physiological pH, the MUC5B construct used in this study (Figure 1B) also contains a titratable histidine residue toward the C-terminal end. With the p*K*_a_ of the imidazole ring of histidine (∼6) lower than physiological pH (∼pH 7.4),^70^ we can assume that histidine will predominantly exist in its deprotonated form and have therefore applied the MARTINI 3 mapping for a neutral histidine residue. A representative example of CG mapping applied to the glycopeptides in this study is shown in Figure 2. This is shown for the G1 glycopeptide from the Mini2 MUC5B structure from Kearns et al.^10^ (Figure 1C), which covers the majority of the bead types found in the other 17 glycopeptides. The full mapping is shown in the Supporting Information (Figure S2).

**Figure 2:**
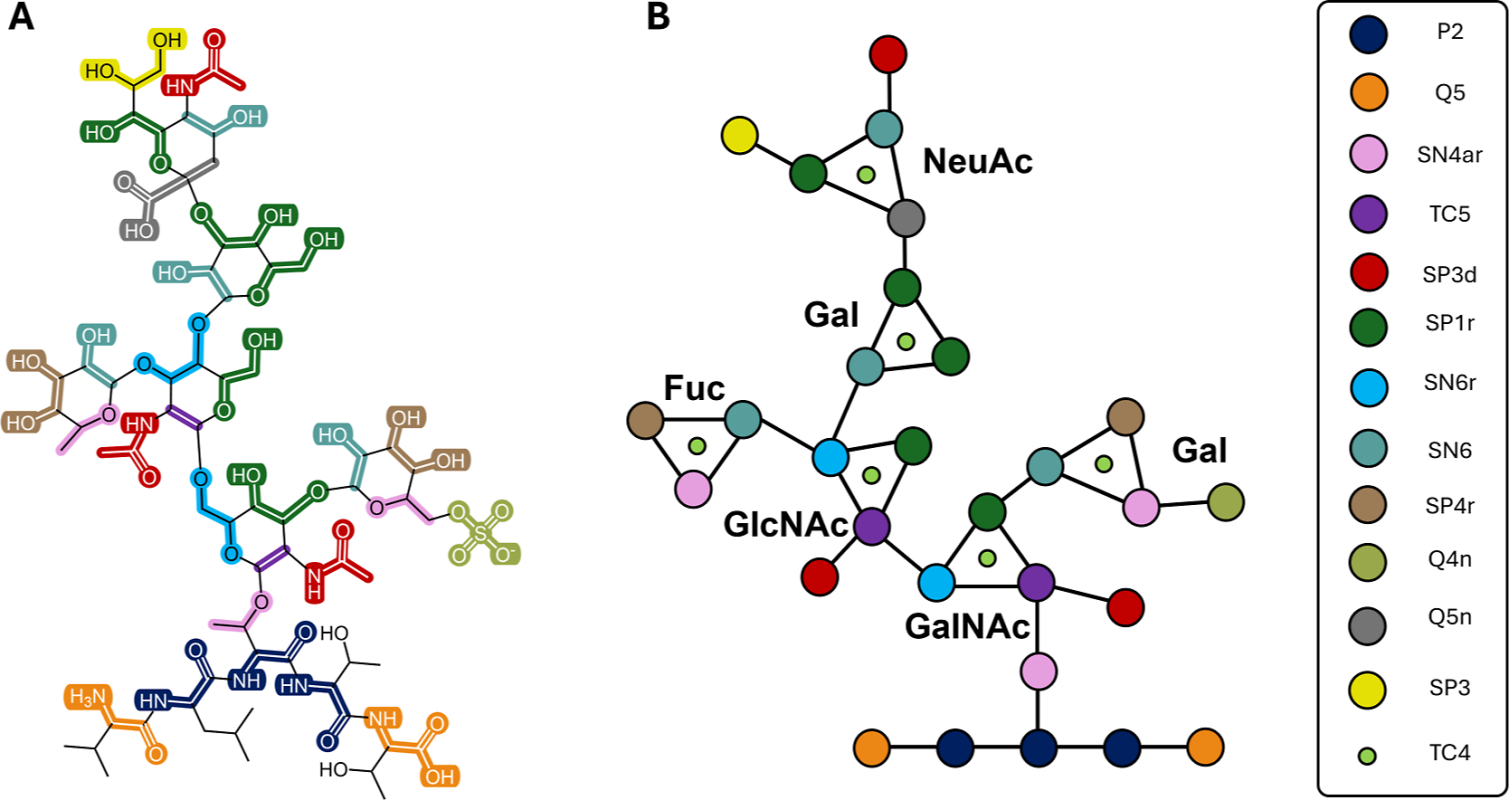
CG MARTINI 3 mapping of a representative glycopeptide. (A) Atomistic representation of G1 glycopeptide, colored according to their corresponding CG bead type. (B) CG representation of G1 glycopeptide. The side chain beads of the first two and last two amino acids followed the standard mapping recommended in MARTINI 3 ^24^ and have been omitted in (B) for simplicity. A key illustrating the different coarse grained bead types used in the mapping is also provided.

### Coarse-Grained Parameterization

The atomistic trajectories of the 18 glycopeptides were subsequently used to generate CG MARTINI-bonded parameters, specifically those concerning bond stretching, angle bending, and dihedral twisting. We used the PyCGTOOL^66^ package to implement a modified Boltzmann inversion method^71^ on the bonded distributions throughout the atomistic MD trajectories. The LAMMPS atomistic trajectories were passed to PyCGTOOL as GROMACS files, with the Visual Molecular Dynamics (VMD) software^72^ being used to convert the LAMMPS trajectories into the GROMACS file format.

To validate the bond parameters of the glycans, the resulting parameters and the CG topology files from PyCGTOOL were used to set up CG simulations for each of the 18 glycopeptides, with the PyCGTOOL files initially converted into Moltemplate text files.^73^ A larger system size (box length of 80 Å) was utilized for the CG simulations as compared to the all-atom simulations (box length ≤ 50 Å), due to preliminary simulations of smaller CG systems illustrating large temperature fluctuations. Each system was solvated with Martini 3 water beads (W), where a single water bead represents four water molecules. The number of water molecules added to each system was determined by subtracting the total volume occupied by the glycopeptides, as determined using the GROMACS gmx solvent accessible surface area (SASA) software,^74–76^ from the volume of the box. The remaining volume was filled with the appropriate number of water beads to achieve a density of 1 g cm^–3^. Martini 3 TQ5 beads representing sodium cations and chloride anions beads were also added to neutralize the system and achieve a background salt concentration of 150 mM. The exact composition of the CG systems for the 18 individual glycopeptides is further outlined in the Supporting Information (Table S1). The initial configuration of the solvated and neutralized system was generated using the PACKMOL software,^77^ which in conjunction with the Moltemplate parameter text files and the Moltemplate software^73^ was used to generate the LAMMPS data files.

Bond stretching within the CG model was represented using a harmonic potential. Consistent with previous MARTINI simulations,^29,78–80^ dihedral potentials can lead to numerical instabilities, and preliminary simulations of the glycopeptides under the NVE ensemble were found to exhibit poorer energy conservation upon implementing dihedral potentials. Thus, as recommended by Grünewald et al.,^28^ the restricted bending potential,^81^ which stops the angles from reaching 180°, to prevent numerical instabilities due to the calculation of the dihedral angles, was implemented. This enabled a time step of 5 fs to be used for the MARTINI 3 simulations while also modeling the dihedral terms, which was described using a periodic dihedral potential. Unlike previous MARTINI parameterizations for *N*-glycans^29^ and globular proteins,^50^ matching the angle distributions from the atomistic simulations did not require the use of elastic networks or Go̅-like models.

The MARTINI 3 model of saccharide structures by Grünewald et al.^28^ and Brandner et al.^64^ implements the LINCS algorithm^82^ to constrain the bonds within the rigid CG triangular structures. Although the LINCS algorithm is available from the GROMACS software,^75,76^ it is not available in LAMMPS,^52^ and thus we instead treat each monosaccharide unit as a rigid body using the fix rigid command.^83^ Rigid constraints can lead to numerical instabilities, particularly for extended networks.^68^ However, we observe that the use of rigid bodies coupled with the CG nature of our systems enabled a time step of 5 fs. Furthermore, the MARTINI 3 mapping of monosaccharides^28^ makes extensive use of virtual sites, specifically with a TC4 bead, to improve stability and enforce geometrical features such as ring planarity.^84^ In order to implement the virtual sites in our LAMMPS simulations, we have modeled the TC4 virtual site as a particle of negligible mass (1 × 10^–100^ Da), with the application of the rigid body constraint maintaining the position of the virtual site at the center of geometry of the triangular framework of each CG monosaccharide. Each system underwent an initial energy minimization and was subsequently equilibrated in the *NVT* ensemble, initially for 25 ps (Δ*t* = 0.25 fs), followed by a longer equilibration of 25 ns (Δ*t* = 5 fs). Production run simulations under the *NVT* ensemble were performed for a cumulative time of 500 ns, with a time step of 5 fs. The rigid bodies^83^ and the non-rigid molecules underwent separate integration and thermostatting at 300 K using the Nosé–Hoover thermostat.^54,55^ Thus, both the equilibration and production runs were performed under the canonical ensemble (*NVT*) due to the inability to adjust the volume of the system with more than one integrator and barostat. A cutoff of 1.2 nm was implemented for the short-range electrostatic interactions, with long-range electrostatic interactions beyond this cutoff calculated using the PPPM algorithm.^59,60^ The Lennard-Jones contributions were also treated with a cutoff of 1.2 nm, with the LJ interactions smoothly shifted to zero from 0.9 to 1.2 nm. During both the equilibration and production run of all CG simulations, a weak tether was applied to the glycopeptide using the fix spring command to overcome a drift in the center of mass of the system. The simulations in LAMMPS required a few adjustments to how the MARTINI 3 force field is commonly implemented with GROMACS,^75,76^ as has been detailed above. Therefore, for a subset of systems, we validate our LAMMPS simulations against preliminary simulations performed with the GROMACS software.^75,76^

Distributions of the CG bonded parameters throughout the MD simulations were determined using the PyCGTOOL software.^66^ The parameters were subsequently validated through comparisons of the distributions against the respective bond distributions from the atomistic MD simulations. Further validation of the CG parameters of the glycopeptide was performed by measuring the radius of gyration throughout the CG and atomistic simulations using the MDAnalysis package.^85,86^ In addition, comparisons of the molecular volume and geometry of the CG model against the atomistic model via the determination of the SASA are also performed.^27,28,64,87^ We calculated the SASA using the double cubic lattice method^74^ in GROMACS,^75,76^ and we compared the SASA of the 18 glycopeptides to the atomistic MD simulations with CHARMM36m. A probe radius of 0.191 nm was used for the SASA calculation for both the atomistic and CG simulations. The van der Waals radii used for the atomistic simulations were based on the values from Rowland and Taylor,^88^ while the SASA calculations for the CG simulations employed a vdW radii of 0.191 nm, 0.230 nm, and 0.264 nm for the tiny, small, and regular beads, respectively.

### Coarse-Grained MD Simulations of MUC5B

Following validation of bonded parameters, the PyCGTOOL topology files of the 18 glycopeptides were overlaid and merged with each other using the PyMOL software,^62^ yielding a CG topology file of the 30-amino acid glycopeptide. The MARTINI 3 CG mapping for the Mini2 structure of MUC5B from Kearns et al.^10^ is illustrated in Figure 1F, with a larger version shown in the Supporting Information (Figure S2). Along with the topology coordinates, the bond, angle, and dihedral parameters of each glycopeptide were merged and added to Moltemplate files, which were then converted into LAMMPS files using the Moltemplate software.^73^ With our 18 smaller glycopeptides each comprising a short segment of the protein backbone (5 amino acids), we therefore utilized the Martinize2 software^89^ to generate optimized parameters of the whole 30 amino-acid protein backbone. The DSSP secondary structure of the protein backbone was set to C (coil) in the Martinize2 software,^89^ with no elastic network applied to the protein to reduce the effect of constraining the protein backbone.

The CG model was solvated in a cubic box of length 125 Å, with 15,860 water (W) beads added, while 13 TQ5 sodium cation beads were added to neutralize the charged glycopeptide system. An additional 171 TQ5 sodium cation beads and 171 TQ5 chloride anion beads were added to achieve a background NaCl concentration of 150 mM. As with the previous CG simulations carried out in this study, the initial configuration of the solvated and neutralized system was determined by using the PACKMOL software.^77^ For the nonbonded LJ interactions, we use standard MARTINI 3 ^24^ parameters for proteins.^90^ In MD simulations with MARTINI 2, it has become common to downscale the protein–protein LJ interactions relative to the protein–water and water–water interactions to avoid excessive aggregation.^80,91,92^ It has recently been shown that reducing the strength of protein–protein LJ interactions by around 10% also leads to improved agreement with experiments for a wide range of IDPs in MARTINI 3.^25,26^ Since mucins contain an IDP backbone,^10^ we also investigate the effect of downscaling the protein–protein LJ interactions, reducing the ε value of the LJ potential by a factor of 0.88.^26^ The single MUC5B glycopeptide system underwent an energy minimization, followed by an initial equilibration under the *NVT* ensemble for 25 ps (Δ*t* = 0.25 fs) and a subsequent longer *NVT* equilibration of 12.5 ns (Δ*t* = 5 fs). The production run simulations were run under the *NVT* ensemble for a cumulative time of 250 ns, with a time step of 5 fs.

Structural properties, including the radius of gyration, *R*_g_, and end-to-end distance, *R*_ee_, were calculated using the MDAnalysis software.^85,86^ The radius of gyration is commonly compared to experiments and atomistic simulations for the validation of CG MARTINI parameters of polymers^93–97^ and biomacromolecules.^25,26,28,78^ Therefore, we compare the *R*_g_ of the 18 short glycopeptides and the 30-amino acid glycopeptide with MARTINI 3 to atomistic simulations with CHARMM36m.^48^ A power-law scaling of the *R*_g_ with molecular weight, *M*_w_, is frequently observed that can be directly compared to experiments.^93,96,97^ This gives an indication of the conformation of the polymer (or protein) and the stiffness of the backbone. We replicate the 30-amino acid consensus sequence to produce glycoproteins of higher molecular weight, with two (60-amino acids) to five (150-amino acids) repeats being simulated. The starting conformations of the repeated mucin structure were initially determined by replicating the structures on PyMOL.^62^ Each system was then solvated with the number of water molecules determined using the same procedure as outlined previously. Sodium cation and chloride anion beads were added to neutralize the system and achieve a salt concentration of 150 mM. The systems containing the 2–5 repeats underwent an energy minimization, an initial equilibration under the *NVT* ensemble for 25 ps (Δ*t* = 0.25 fs), followed by a subsequent *NVT* equilibration of 25 ns (Δ*t* = 5 fs). Production run simulations were run for a cumulative time of 500 ns with the radius of gyration measurements taken from the last 200 ns. The exponent of the power-law scaling of the *R*_g_ with *M*_w_ was then compared with the scaling relationship measured for mucins of higher molecular weight from previous experiments. In addition to assessing the power-law scaling of the radius of gyration of the glycosylated PTS domain with its molecular weight, we also assessed the effect of removing the glycans on the scaling relationship. This entailed simulating one (30-amino acids) to six (180-amino acids) repeat units of the unglycosylated PTS domains, with the initial coordinates of the repeats generated through implementing a random walk algorithm using the Polyply package.^98^ The simulations of the unglycosylated MUC5B systems also comprised an energy minimization and equilibration in the *NVT* ensemble, followed by *NVT* production runs performed for a total simulation time of 500 ns. The system setups for both the glycosylated and unglycosylated MUC5B systems are further outlined in the Supporting Information (Table S2).

### Atomistic MD Simulations of MUC5B

Atomistic simulations of the 30-amino acid glycopeptide were also performed to validate our MARTINI 3 parameters for MUC5B. We followed a protocol similar to that used for the atomistic simulations of the smaller glycopeptides. The 30-amino acid backbone structure was predicted using AlphaFold 3,^39,40^ and CHARMM-GUI^41–45^ was used to glycosylate the protein backbone. CHARMM-GUI^41–45^ was then used to solvate the system using 43997 TIP3P water molecules,^49^ with 13 sodium counterions added to neutralize the system. An additional 125 sodium and 125 chloride ions were also added to achieve a 150 mM background salt concentration. The solvated glycopeptide was subsequently parameterized with the CHARMM36m parameters^48^ using the CHARMM-GUI software.^41–45^ The MUC5B protein system was initially energy minimized, after which it was subject to a short equilibration in the *NVT* ensemble for 20 ps and a longer equilibration in the *NPT* ensemble for 5 ns. The equilibrated system subsequently underwent production run simulations in the *NVT* ensemble for a cumulative time of 250 ns, with a time step of 2 fs.

## Results and Discussion

### Bonded Parameter Validation

The bonded parameters of the glycans developed in this study were validated by comparing the distributions of the bonded terms during atomistic and CG simulations of the glycopeptides. Figure 3 illustrates representative comparisons of the probability distribution functions of selected bonds, angles, and dihedrals for the atomistic (CHARMM36m^48^) and CG (MARTINI 3 ^24^) MD simulations of the G1 glycopeptide. Additional comparisons of the distributions are provided in the Supporting Information for the G1 (Figure S3), G2 (Figure S4), G3 (Figure S5), and G14 (Figure S6) glycopeptides, the latter of which is an example of a serine-linked *O*-glycan, while the former three figures are examples of the more abundant threonine-linked *O*-glycan. Parity plots comparing all of the mean values of the bond lengths (Figure S7) and bond angles (Figure S8) between the atomistic and CG MD simulations are also illustrated in the Supporting Information. Overall, there is good agreement between the atomistic and CG descriptions for the bonds, angles, and dihedrals. The absolute percentage deviation between the atomistic and CG description ranges between 0.0 and 6.1% for the mean bond distances and 0.0 and 22.86% for the mean bond angles, with a median percentage difference of 0.19 and 1.44% observed for the mean bond distances and angles, respectively. With many of the dihedral distributions exhibiting multimodal behavior, the mean value in this case did not provide a meaningful basis for validating the dihedral parameters between the atomistic and CG simulations. Although the majority of the dihedral parameters derived in this study was shown to largely reproduce the multimodal distributions observed with the dihedral terms from atomistic simulations (Figures S3–S6C), a small number of CG dihedral parameters did not capture the multimodal behavior (e.g., second plot of Figure S3C). These discrepancies may be attributed to the limited representation of both the steric effects of neighboring hydroxyl groups and the effects of intramolecular hydrogen bonding on the orientation of the glycosidic linkage upon coarse-graining atoms into beads. While this may be addressed by applying a finer CG MARTINI approach through utilizing the T (“Tiny”) beads to a greater extent than has currently been employed in this study, particularly in regards for the hydroxyl groups neighboring the glycosidic bonds, this would likely offer only a marginal improvement.

**Figure 3:**
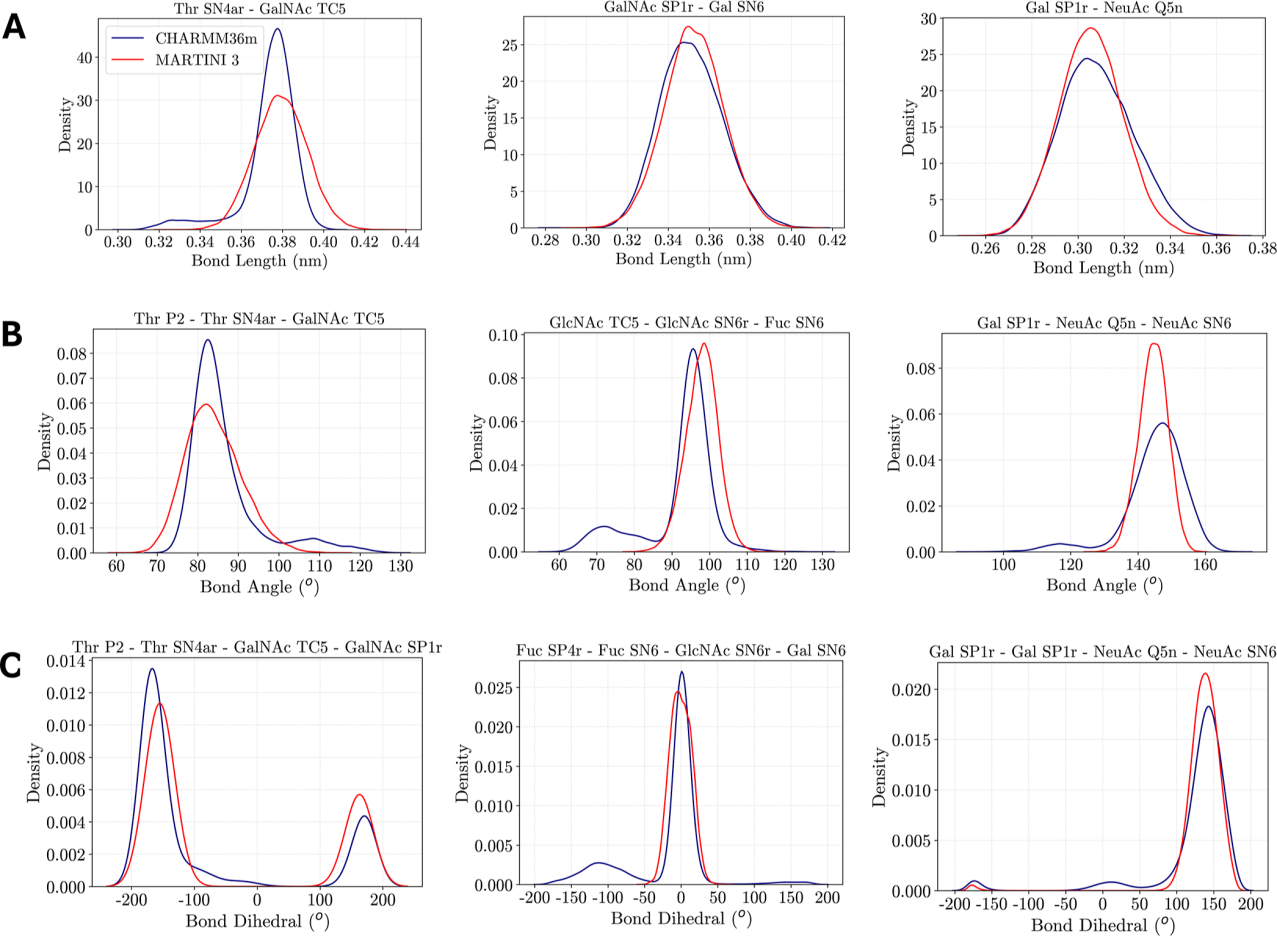
Distributions of selected bond lengths (A), angles (B), and dihedral angles (C) within the G1 glycopeptide from atomistic MD simulations with CHARMM36m^48^ (blue) and CG MD simulations with MARTINI 3 ^24^ (red).

We did also observe narrow distributions for bonds within the individual constituent monosaccharide structures, as is shown in the plot furthest to the right of Figure S3A in the Supporting Information. These observations are a result of our model treating the individual sugars as rigid bodies^83^ as opposed to using the LINCS algorithm^82^ to constrain the bonds, which is not available through the LAMMPS simulation package. To validate our approach of modeling MARTINI 3 CG sugars with the rigid body constraints using the LAMMPS software,^52^ we have also compared our observed distribution of the bonded terms with simulations performed with the GROMACS software^75,76^ for the G1 glycopeptide, where the LINCS algorithm^82^ was applied to the sugars. As illustrated in Figure S3 in the Supporting Information, there is excellent agreement between the MARTINI 3 simulations performed in LAMMPS (red) and GROMACS (green), with both simulations aligning well with the atomistic simulations performed in LAMMPS. Furthermore, the distributions of bond distances within the individual constituent sugars during the GROMACS CG simulations were also found to align well with the atomistic simulations.

In addition to validating the bonded parameters, the structural properties of the 18 glycopeptides were also compared between the atomistic and CG simulations. A good agreement was found between the mean radius of gyration of the individual glycopeptides from the CHARMM36m and MARTINI 3 simulations, as shown in the parity plot in Figure 4A. The percentage deviation of the mean radius of gyration observed between the atomistic and CG simulations ranged from 0.2% (G13 glycopeptide) to 3.7% (G1 glycopeptide). The SASA of the small glycopeptides was also compared between those of the atomistic and CG simulations (Figure 4B). The CG simulations were found to slightly underestimate (0.7–6.2%) the SASA compared to the atomistic simulations. Underpredicted SASA values (∼8%) were also previously observed for carbohydrates parameterized with the MARTINI 3 force field.^28^ In the aforementioned study, improved agreement with atomistic SASA values was obtained by increasing the bond lengths within the sugar rings by 15%,^28^ as was previously employed in MARTINI 2.^92^ Since the percentage difference between the mean SASA values of our atomistic and CG glycopeptides remains relatively low, we chose to keep the bond lengths at their default MARTINI 3 values, aligning with the recent parameterization of disaccharides with the MARTINI 3 force field by Brandner et al.^64^

**Figure 4:**
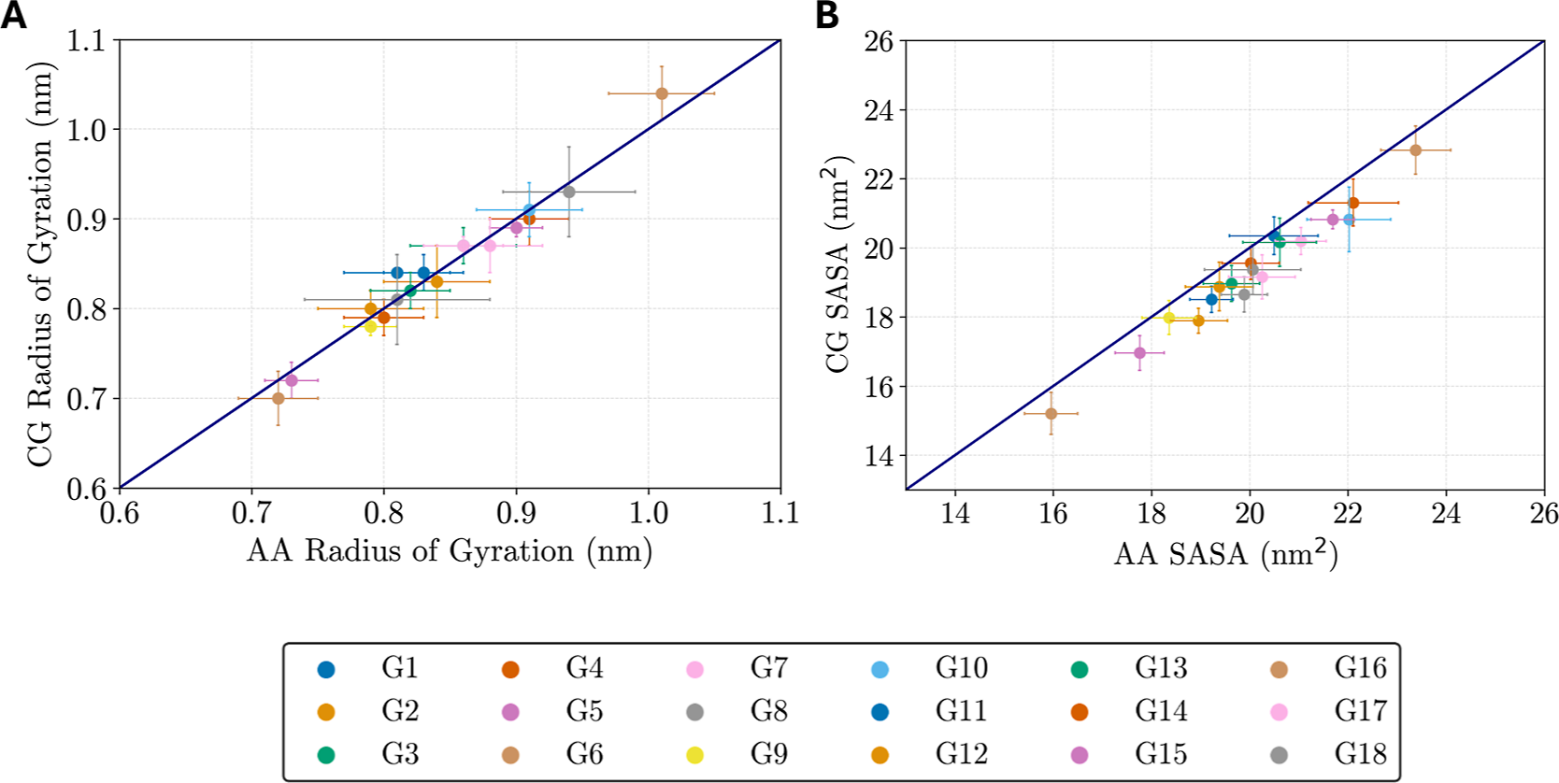
Comparison between the atomistic and CG values of the radius of gyration (A) and SASA (B) for the 18 glycopeptides. Error bars representing the standard deviation are shown.

### Small Glycopeptide

The validated parameters were subsequently used to model the 30-amino acid MUC5B glycopeptide shown in Figure 1F. Comparisons of the time evolutions of the root-mean-square deviation (RMSD) of a pseudo-CG representation^66^ of the CHARMM36m^48^ atomistic model and the CG model using both the default MARTINI 3 parameters^24^ and downscaled protein–protein LJ interactions^26^ are shown in the Supporting Information (Figure S9). The RMSD plots revealed that a similar conformation is attained in the atomistic and CG models. Agreement between the atomistic and CG descriptions was best for the downscaled MARTINI 3 parameters, with both RMSD values stabilizing at ∼1.5 nm. To further assess the structural properties of our CG model, comparisons of the probability distribution functions of different structural properties, including the radius of gyration (*R*_g_), end-to-end distance (*R*_ee_), and solvent-accessible surface area (SASA), for the atomistic and CG MD simulations were also assessed as shown in Figure 5. There is good agreement between the atomistic and CG force fields for all structural properties, with both the default and downscaled LJ interactions. As expected, downscaling the protein–protein LJ interactions leads to a more expanded conformation, although the change for MUC5B is much smaller compared to the unglycosylated IDPs for which previous comparisons have been reported.^26^ This is because the MUC5B glycopeptide adopts an extended bottlebrush conformation,^10^ which is governed more by the electrostatic repulsion between the negatively charged glycans^21^ than the strength of the attractive Lennard-Jones interactions. The downscaling of the protein–protein LJ interactions (*R*_g_ = 2.36 ± 0.06 nm) resulted in a marginal improvement in the agreement of the mean radius of gyration with the atomistic simulations (*R*_g_ = 2.45 ± 0.10 nm) compared to the standard MARTINI 3 parameters (*R*_g_ = 2.30 ± 0.05 nm), as shown in Figure 5A. The mean end-to-end distance was also in good agreement between the atomistic and CG simulations. The CG simulations with downscaled protein–protein interactions (*R*_ee_ = 6.35 ± 0.53 nm) again showed slightly better agreement in the mean end-to-end distance with the atomistic simulations (*R*_ee_ = 6.52 ± 1.12 nm) than the unscaled case (*R*_ee_ = 7.25 ± 0.30 nm). Despite the lower end-to-end distance of the glycoprotein backbone during the CG simulations performed with the downscaled protein–protein interactions compared to the original MARTINI 3 parameters, it is apparent from visualizing the simulations that the entire glycoprotein from the downscaled simulations adopted a more elongated conformation (Figure 5D), aligning more closely to the conformation observed in the atomistic simulations. For MUC5B, the downscaling of the protein–protein self-interactions is likely to have resulted in the protein backbone becoming more flexible due to weaker self-interactions, enabling the protein to potentially explore conformations with a lower end-to-end distance, while also allowing the glycans to project further away from the protein backbone, and thus yielding a more expanded conformation. The spatially expanded structure visualized from the CG simulations when downscaling the protein–protein interactions compared to the original MARTINI 3 parameterization is reflected through an increase in the mean SASA value from 156.7 ± 2.6 nm^2^ to 162.8 ± 3.1 nm^2^, as shown in Figure 5C. The higher mean SASA value observed with the downscaled simulations is also closer to the mean value observed from the atomistic simulations (171.1 ± 5.9 nm^2^).

**Figure 5:**
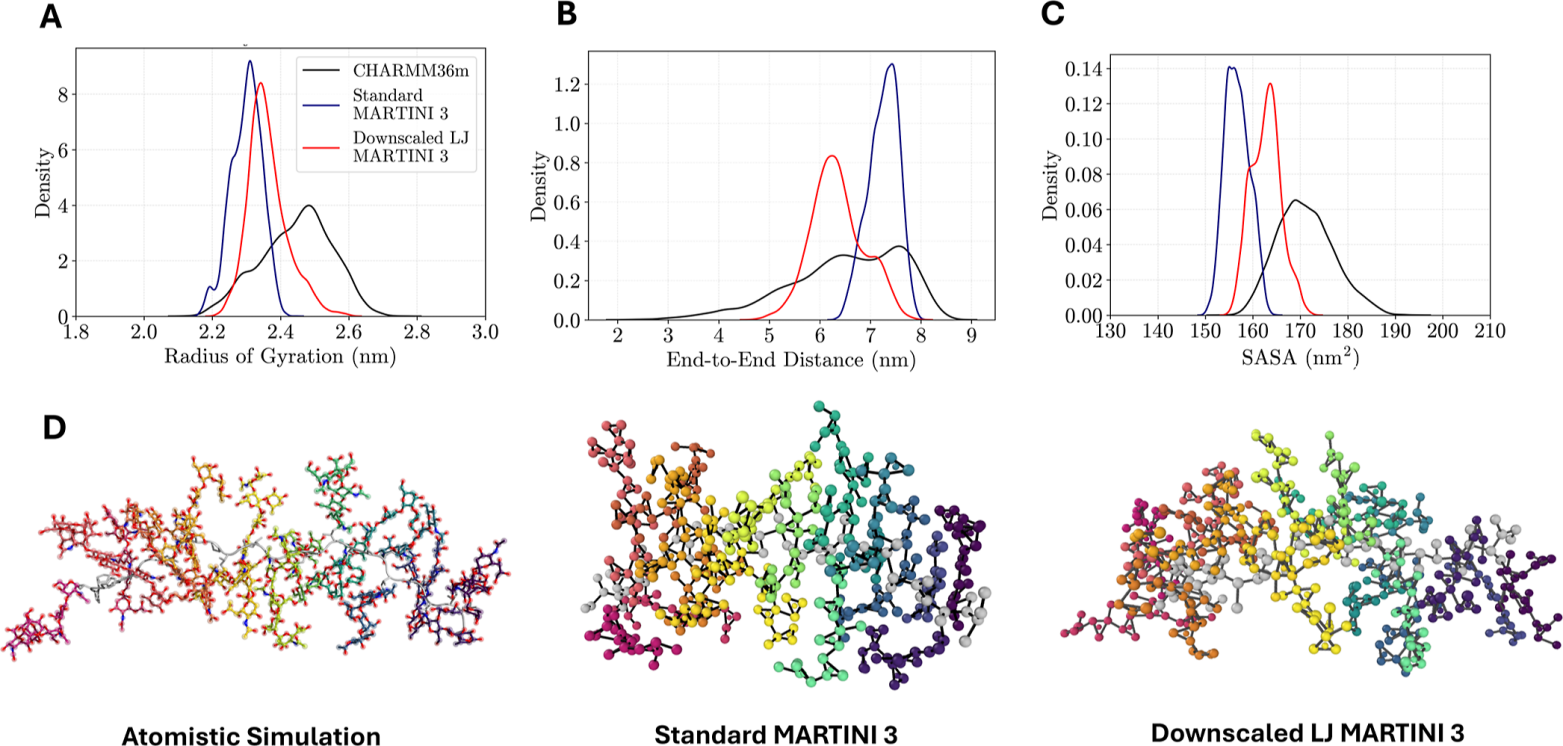
Structural properties of the CG 30-amino acid MUC5B model. Comparative distributions of the radius of gyration (A), end-to-end distance (B), and SASA (C) between atomistic simulations with CHARMM36m^48^ (black), CG simulations performed with original MARTINI 3 parameters^24^ (blue), and CG simulations performed with protein–protein LJ interactions downscaled by a factor of 0.88 ^26^ (red). (D) Conformations of MUC5B after 250 ns MD simulations of the atomistic model, the CG model using the original MARTINI 3 protein–protein LJ interactions and the CG model using the downscaled protein–protein LJ interactions, rendered using PyMOL^62^ and OVITO.^63^ The protein backbone is depicted in gray, and each glycan is denoted with a different color.

It is worth noting, however, that all the three structural properties of MUC5B shown in Figure 5A–C were found to display a wider distribution in the atomistic simulations than in the CG simulations, suggesting a more restricted conformation for the latter. Agreement of all three structural properties could potentially be further improved using recent adaptions to the MARTINI 3 force field. For example, the MARTINI3-IDP^99^ force field optimizes the bonded interactions within the IDP regions of proteins, rather than uniformly downscaling the nonbonded interactions,^25^ as has been employed in this study. Furthermore, the introduction of Go̅ virtual sites to the backbone beads of the protein and the addition of LJ interactions between the Go̅ sites and water beads has also been found to be an effective way to accurately reproduce the conformational properties of other proteins.^100^ While we appreciate both these methods may overcome the small discrepancies observed between the structural properties from the atomistic and CG models, subsequent validation to experimental data, as detailed below, suggests the currently employed parameters accurately capture the structure and dynamics of the glycosylated domains of MUC5B.

Previous MARTINI-based MD simulations of *N*-glycans,^29^ glycosaminoglycans,^101^ and other carbohydrates^28,80^ tend to overestimate their self-aggregation propensity. The glycans in our simulations are bound to the PTS-rich backbone rather than being free in aggregate in solution. However, if the self-interactions between the glycans are too strong relative to those with the solvent, a collapsed mucin conformation may be observed, rather than the expected bottlebrush.^10^ To determine the level of aggregation of the glycans in our simulations, we computed the radial distribution function (RDF) of each glycan with respect to the surrounding glycans on the protein throughout the atomistic simulations and the CG simulations performed with both the standard MARTINI 3 and downscaled protein–protein LJ interactions. Figure 6 illustrates the RDF profile of three *O*-glycans relative to their surrounding glycans, with reasonable agreement observed across the atomistic and CG simulations. Notably, the RDF curves illustrate the presence of a single broad peak at a distance of ∼10 Å, which slowly decays to zero at a distance of ∼60 Å, with similar RDFs been reported for *N*-glycans in solution in previous MARTINI simulations.^29^

**Figure 6:**
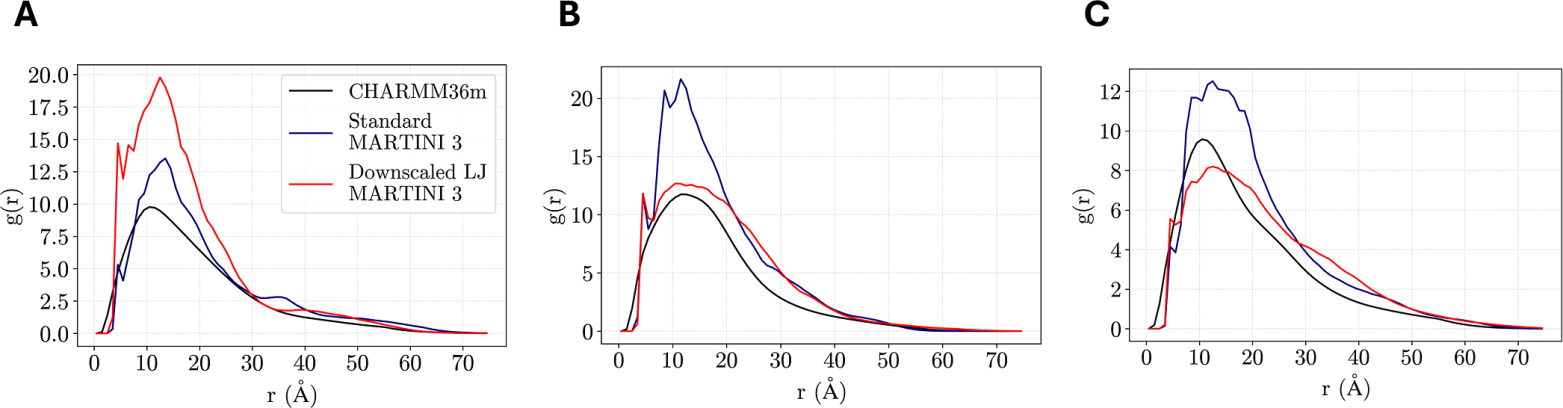
Radial distribution functions of glycans, G2 (A), G14 (B), and G17 (C), with respect to their surrounding glycans compared between the atomistic simulations with CHARMM36m^48^ (black) and CG simulations performed with the original MARTINI 3 ^24^ (blue) and downscaled protein–protein LJ interactions^26^ (red).

Scaling the protein–protein interactions was found in certain instances to reduce the height of the RDF peak found at ∼10 Å, as is illustrated with the RDF curves concerning Glycans, G14 and G17 (Figure 6B,C). By contrast, an opposite effect of scaling protein–protein interactions was noted with the RDF profile of Glycan G2 (Figure 6A). The inconclusive behavior of scaling protein–protein LJ interactions on the aggregation propensity of the glycans further alludes to the marginal influence of protein–protein LJ interactions on the spatial distribution of the glycans on the protein. Rather, it is more likely that the electrostatic repulsions between the negatively charged sugars govern the spatial correlation between the glycans and prevents overaggregation of the glycans when they are attached to the protein backbone. Nevertheless, we can conclude from the RDF curves that the CG MARTINI 3 simulations broadly reproduce the bottlebrush structure of a 30-amino acid MUC5B consensus sequence from atomistic simulations^10^ whether or not the protein–protein interactions are downscaled, with the bottlebrush structure being a commonly observed attribute of mucins in experimental studies.^102,103^ The preservation of the mucin’s bottlebrush structure and the minimal aggregation of the sugars is further complemented by the close alignment of the radius of gyration of each CG glycan on the densely glycosylated 30-amino acid MUC5B protein with their respective atomistic reference values (Figure S10).

### Larger Glycoproteins

Several experimental studies have observed power-law scaling of *R*_g_ with *M*_w_ for mucins, including MUC5B,^11,104,105^ as shown in Figure 7. There is considerable variability in experimental results both for the values of *R*_g_ and the scaling exponent, *v*, which from Flory theory is given by R_g_ = *R*_0_ *M*_w_^v^, where *R*_0_ is a constant.^106^ In a recent study, Hughes et al.^11^ measured *R*_g_ values of human salivary MUC5B between ∼109.6 and 167.8 nm for molecular weights between ∼5.77 and 13.6 MDa, with a Flory scaling exponent of *v* = 0.505. They also found that, although adding calcium salts reduced the *R*_g_, the reduction was consistent across the various molecular weights studies and thus the scaling exponent remained unaffected.^11^ Previous studies by the same group^105^ also assessed the power-law scaling *R*_g_ with *M*_w_ for equine airway mucin of similar molecular weights (2–20 MDa), with *R*_g_ values between 62 and 270 nm, giving rise to a slightly higher scaling exponent of *v* = 0.688. A larger scaling exponent of *v* = 0.780 has also been observed with lower molecular weight human airway MUC5B (∼0.45–0.88 MDa) by the same group.^104^ This wide range of experimental scaling exponents for MUC5B spans those expected for random coils (*v* = 0.5–0.6) and extends towards the value for rigid rods (*v* = 1.0).^107^ Given the large size of MUC5B,^10^ we cannot directly compare our simulation *R*_g_ results for the entire glycoprotein. Instead, we compare the Flory scaling exponent obtained from simulations with different sizes of PTS-rich glycopeptides to the experimental values. Since the glycans in mucins account for around 80% of their total molecular weight,^7^ the glycosylated domains will largely govern the conformation of the protein. Quantitative agreement between the simulations and experiments cannot be expected given that we do not simulate other parts of the protein, namely, the vWF-like C- and N-terminal and cysteine-rich domains. These regions have compact globular conformations that contain stacked β-sheet structures.^13^ Thus, it is expected that the simulations, which only consider the disordered glycosylated domains, will overestimate the scaling exponent compared to experiments.

**Figure 7:**
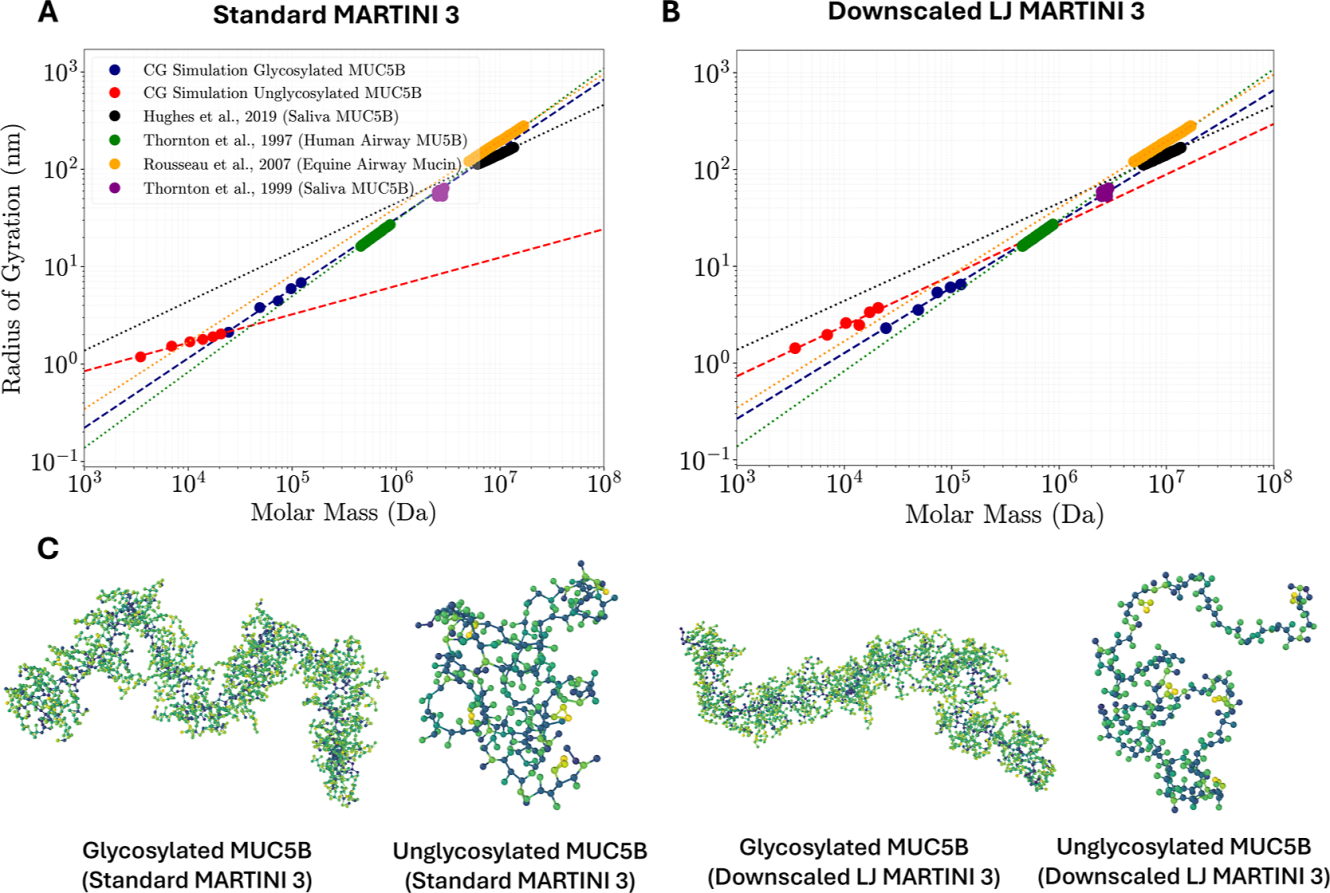
Scaling of the radius of gyration with molar mass for glycosylated (blue) and unglycosylated (red) CG models of MUC5B. Power-law fits to both the experimental data (dotted lines) and our simulation data (dashed lines) are illustrated with the scaling exponent, *v*, given below. Results are shown from simulations with the standard MARTINI 3 protein–protein LJ parameters^24^ (*v* = 0.715) (A) and protein–protein LJ interactions downscaled by a factor of 0.88 ^26^ (*v* = 0.679) (B). The results are compared with experimental data from Hughes et al.^11^ (black, *v* = 0.505), Thornton et al.^104^ (green, *v* = 0.780), Rousseau et al.^105^ (orange, *v* = 0.688), and Thornton et al.^108^ (purple). (C) Conformations of a 150-amino acid glycosylated and unglycosylated CG MUC5B structure after 500 ns MD simulations using the standard and downscaled MARTINI 3 protein–protein LJ parameters, rendered with OVITO.^63^

Using the standard MARTINI 3 LJ interactions, our MD simulation results for the glycosylated PTS-rich domain of MUC5B give a scaling exponent *v* = 0.715, as shown in Figure 7A. This suggests a relatively rigid protein with a scaling exponent between those expected for rigid rods (*v* = 1.0) and random coils (*v* = 0.5–0.6).^107^ The scaling exponent from standard MARTINI 3 is towards the top end of the range of the experimental values, close to that reported by Thornton et al.^104^ The PTS-rich mucin backbone is IDP,^21^ and standard MARTINI 3 has been shown to yield conformations of IDPs that are too compact.^25^ Downscaling the protein–protein LJ interactions has been shown to improve agreement with experimental results for IDPs.^25,26^ When the MARTINI 3 protein–protein interactions are downscaled by a factor of 0.88, our MD simulation results with MARTINI 3 gives a scaling exponent *v* = 0.679, as shown in Figure 7B. Extended simulations of 1 μs were also performed with the GROMACS software (Figure S11), with scaling exponents of 0.674 and 0.702 observed for the downscaled and standard MARTINI 3 simulations, respectively, closely aligning with those observed from the LAMMPS simulations. It is therefore apparent from both the LAMMPS and GROMACS simulations that the downscaling of the protein–protein LJ interactions was found to reduce the scaling exponent, which more closely aligns with the experiential values reported by Rousseau et al.^105^ and Hughes et al.,^11^ but is further from the larger value reported by Thornton et al.^104^ While downscaling the LJ interactions leads to a slightly larger radius of gyration for the shortest MUC5B system (Figure 5A), the radius of gyration decreases slightly compared to that of the standard MARTINI 3 interactions for the longer chains. This observation confirms that the conformation of MUC5B is governed mostly by repulsion between the negatively charged glycans, rather than LJ interactions between the backbone beads. The effect of downscaling protein–protein LJ interactions with the glycosylated protein is negligible compared to previous simulations of other IDPs.^25,26^ Crucially, the scaling exponent from both our MARTINI 3 simulations with standard and downscaled LJ interactions falls within the experimental range.

The large variation in the experimental values of the scaling exponent, *v*, may be due to differences in the source of the MUC5B samples and how they were stored and prepared. Reduced glycan levels have been reported in mucins extracted from saliva of older patients^109^ and particularly those with dry mouth syndrome.^110^ Structural collapse of mucins upon removal of the glycans is well-known and leads to compaction from an elongated state into a globular conformation.^111–114^ Thus, the lower *v* values observed in some of the experiments, notably Hughes et al.,^11^ could be due to reduced glycosylation. To test this hypothesis, we compared our original MARTINI 3 results with glycopeptides to a subset of simulations of the nonglycosylated PTS-rich backbone, performing simulations ranging from 1 to 6 repeat units of the PTS domain. For the standard MARTINI 3 parameters, we found that the removal of glycans from the glycopeptide substantially reduced the scaling exponent to *v* = 0.292, as shown in Figure 7A. These values imply a collapsed sphere (*v* = 0.33) conformation^107^ and is below that expected for protein backbones (*v* = 0.4).^106^ When the MARTINI 3 protein–protein interactions are downscaled by a factor of 0.88,^26^ the scaling exponent of the unglycosylated PTS-rich backbone is *v* = 0.521, which is still lower than the glycosylated MUC5B but is indicative of a random coil rather than a collapsed conformation. This observation illustrates the stronger effect of downscaling protein–protein LJ interactions for unglycosylated rather than glycosylated IDPs. The effect of downscaling the protein–protein LJ interaction with the unglycosylated protein is further reflected in the more expanded conformations observed at the end of the simulations, as shown in Figure 7C. Notably, the scaling exponent for unglycosylated MUC5B with downscaled LJ interactions was found to be in close agreement with the value from Hughes et al.,^11^ which suggests that reduced levels of glycosylation may have been present in the MUC5B samples used in these experiments.

In addition to the level of glycosylation, another possible point of difference between our simulations and the experiments is the salt concentration, since this has been shown to considerably affect the radius of gyration of mucins.^21^ In our MARTINI 3 simulations, we use a background NaCl concentration of 150 mM, but this can vary considerably (12–217 mM) between individuals in human saliva samples.^51^ Hughes et al.^11^ saw no change in the scaling exponent when adding 10 mM CaCl_2_ to MUC5B. Previous CG simulations^21^ showed that divalent salts such as CaCl_2_ have a greater effect on the radius of gyration than monovalent salts like NaCl. Thus, variations in salt concentrations probably cannot explain the differences in the scaling exponent between the experiments and simulations.

In summary, our CG MARTINI 3 parameters for MUC5B accurately capture various structural properties from atomistic simulations and experiments. A slightly improved agreement is obtained when the protein–protein LJ interactions are downscaled by a factor of 0.88, although the change is marginal compared with unglycosylated IDPs. Our MARTINI 3 model of MUC5B, which follows previous atomistic simulations,^10^ could be made more realistic by including the vWF-like C- and N-termini,^14^ as well as the cysteine-rich domains.^13^ Although these unglycosylated domains make up only a small fraction of the total molecular weight of mucins,^7^ they are important for their self-assembly, gelation, and surface adsorption properties.^12,15^

## Conclusions

We extended the Martini 3 CG force field to mucins. First, we performed atomistic MD simulations of 18 short glycopeptides from the PTS-rich protein backbone and *O*-glycan branches found in salivary mucin MUC5B. We used these simulations with the CHARMM36m force field to parameterize the bonded interactions in MUC5B for the CG MARTINI 3 force field. Our MARTINI 3 parameters reproduced the probability distributions for the bond lengths, bond angles, and dihedral angles of the short glycopeptides from the atomistic simulations. Structural properties of the short glycopeptides, such as the radius of gyration and SASA, are also in good agreement between CHARMM36m and MARTINI 3. We then combined the short glycopeptides to obtain a MARTINI 3 model of a 30-amino acid consensus sequence of MUC5B. Our MARTINI 3 parameters also broadly reproduce the radius of gyration, end-to-end distance, and SASA of the longer 30-amino acid MUC5B glycopeptide from the atomistic model. Downscaling the protein–protein interactions leads to a slightly more expanded conformation and somewhat better agreement with the atomistic simulations. The bottlebrush structure of MUC5B seen in atomistic simulations and experiments is maintained with MARTINI 3, with an analysis of the radial distribution functions confirming that excessive aggregation of the glycans is not observed. We also compared the increase in the radius of gyration with molecular weight to experiments for larger glycoproteins containing up to 150 amino acids. We observe a power-law scaling of the radius of gyration with the molecular weight for MUC5B, with an exponent that is within the range of those reported in previous experimental studies. The scaling exponents observed in the simulations and experiments are between those expected for random coils and rigid rods. Slightly improved agreement with most of the experiments is obtained with downscaled protein–protein LJ interactions. We show that removal of the glycans decreases the scaling exponent as the protein structure collapses. For the standard MARTINI 3 parameters, this leads to a collapsed sphere conformation, whereas the downsized LJ interactions result in a random coil. We expect that these MARTINI parameters will be useful not only for CG MD simulations of MUC5B but also for various other mucins, *O*-glycan-containing proteins such as lubricin, as well as serum and cell membrane glycoproteins.

## Supplementary Material



## Notes

System setups used for the MD simulations of the glycopeptides for the MARTINI 3 parameterization and the MD simulations of the larger glycoproteins for comparison with experiments and figures showing SNFG representation of the MUC5B glycopeptide, full MARTINI 3 mapping of MUC5B, additional comparisons of bonded terms from the atomistic and CG MD simulations, parity plots for all bond lengths and angles for the 18 glycopeptides from the atomistic and CG MD simulations, time evolution RMSD plots during atomistic and CG MD simulations, comparisons of the mean radius of gyration of the CHARMM36m and MARTINI 3 glycans attached to the 30-amino acid PTS domain, and plots illustrating the scaling of the radius of gyration with molar mass for the CG MUC5B model run in GROMACS (PDF)
  The MARTINI 3 parameters developed in this study are available in a public GitHub repository, which can also be found on Zenodo (10.5281/zenodo.17815272). This repository contains the LAMMPS data and input files for the CHARMM36m and MARTINI 3 simulations, along with GROMACS input files for MARTINI 3 simulations.
