# Supplementary material for: A Coarse-Grained MARTINI Model for Mucins
Source: J Chem Theory Comput. 2025 Dec 31;22(1):723–37. doi: 10.1021/acs.jctc.5c01655 (PMC12805576; doi:10.1021/acs.jctc.5c01655)
Supplement: Supplementary file 1 [file ct5c01655_si_001.pdf]

Supporting Information for: A Coarse-Grained MARTINI Model for Mucins

Thilakan Kanesalingam,*,† Erik Weiand,† Philippa M. Cann,† Marc Masen,† and James P. Ewen*,†,‡

†*Department of Mechanical Engineering, Imperial College London, South Kensington Campus, London SW7 2AZ, U.K.*

‡*Department of Mechanical Engineering, University of Bath, Claverton Down, Bath BA2 7AY, U.K.*

E-mail: thilakan.kanesalingam19@imperial.ac.uk; j.ewen@imperial.ac.uk

Table S1: Details of system setup for atomistic and coarse-grained simulations of the 18 glycopeptides, including system size, number of water molecules, Na^+ and Cl^- ions.

Glycopeptides	Atomistic Simulations				Coarse-Grained Simulations			
	Box Length (Å)	Number of TIP3P Water Molecules	Number of Na^+ Ions	Number of Cl^- Ions	Box Length (Å)	Number of Water Beads	Number of TQ5 Na^+ Beads	Number of TQ5 Cl^- Beads
**G1**	46	2909	10	8	80	4240	47	45
**G2**	41	2052	5	5	80	4245	45	45
**G3**	47	3123	10	9	80	4245	46	45
**G4**	48	3342	9	9	80	4240	45	45
**G5**	43	2415	7	6	80	4250	46	45
**G6**	45	2815	7	7	80	4250	45	45
**G7**	49	3560	11	10	80	4240	46	45
**G8**	46	2929	9	8	80	4245	46	45
**G9**	44	2540	8	7	80	4245	46	45
**G10**	50	3739	12	10	80	4240	47	45
**G11**	45	2799	7	7	80	4245	45	45
**G12**	46	2923	9	8	80	4245	46	45
**G13**	45	2769	7	7	80	4235	45	45
**G14**	44	2528	7	7	80	4240	45	45
**G15**	48	3329	9	9	80	4240	45	45
**G16**	48	3321	9	9	80	4230	45	45
**G17**	48	3336	11	9	80	4245	47	45
**G18**	47	3130	10	9	80	4240	46	45

Table S2: Details of system setup for MARTINI 3 MD simulations of unglycosylated and glycosylated MUC5B glycoproteins, ran in LAMMPS, including system size, number of water beads, Na^+ and Cl^- ions.

	Unglycosylated PTS Domain				Glycosylated PTS Domain			
Number of PTS Domain Repeats	Box Dimensions	Number of Water Beads	Number of TQ5 Na^+ Beads	Number of TQ5 Cl^- Beads	Box Dimensions	Number of Water Beads	Number of TQ5 Na^+ Beads	Number of TQ5 Cl^- Beads
**1**	100 Å x 100 Å x 100 Å	8361	90	90	125 Å x 125 Å x 125 Å	15860	184	171
**2**	120 Å x 120 Å x 120 Å	14328	154	154	200 Å x 200 Å x 160 Å	52572	593	567
**3**	135 Å x 135 Å x 135 Å	20390	220	220	275 Å x 275 Å x 230 Å	143787	1591	1552
**4**	145 Å x 145 Å x 145 Å	25250	272	272	350 Å x 350 Å x 300 Å	305448	3350	3298
**5**	170 Å x 170 Å x 170 Å	40780	440	440	400 Å x 400 Å x 350 Å	465400	5091	5026
**6**	190 Å x 190 Å x 190 Å	56990	615	615				

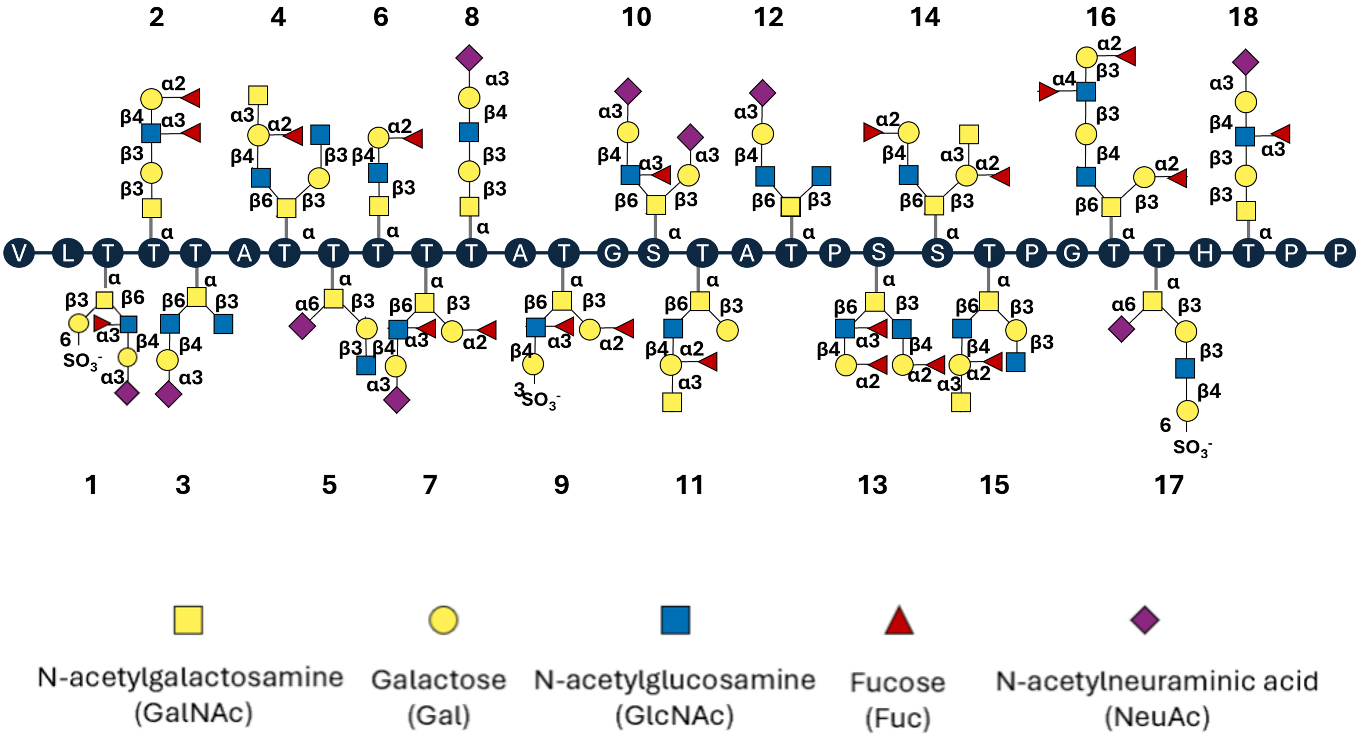

Figure S1: SNFG representation of the MUC5B consensus sequence. A key of the sugars present in the glycans is provided and the glycosidic linkage type is shown in bold. This figure is adapted from Figure 3B(i) of Kearns et al[1] (available under a CC-BY-NC-ND 4.0 License. Copyright 2024 F.L. Kearns, M.A. Rosenfeld and R.E. Amaro).

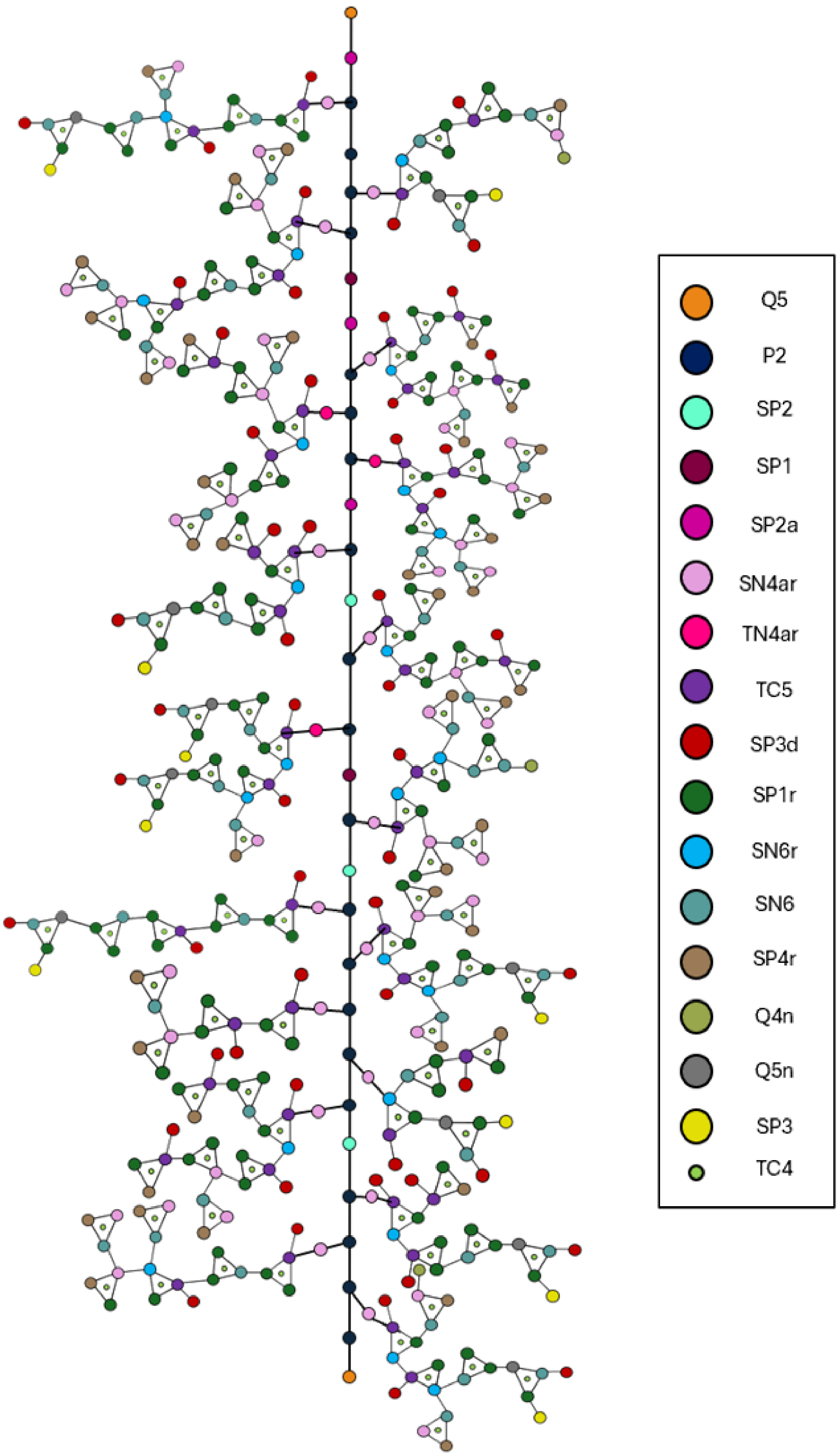

Figure S2: MARTINI 3 Mapping of the MUC5B glycoprotein. A key of the different MARTINI 3 coarse-grained beads is provided. The beads representing the side chains of unglycosylated amino acids were mapped according to the standard MARTINI 3 protein force field[2] and have been omitted from the figure.

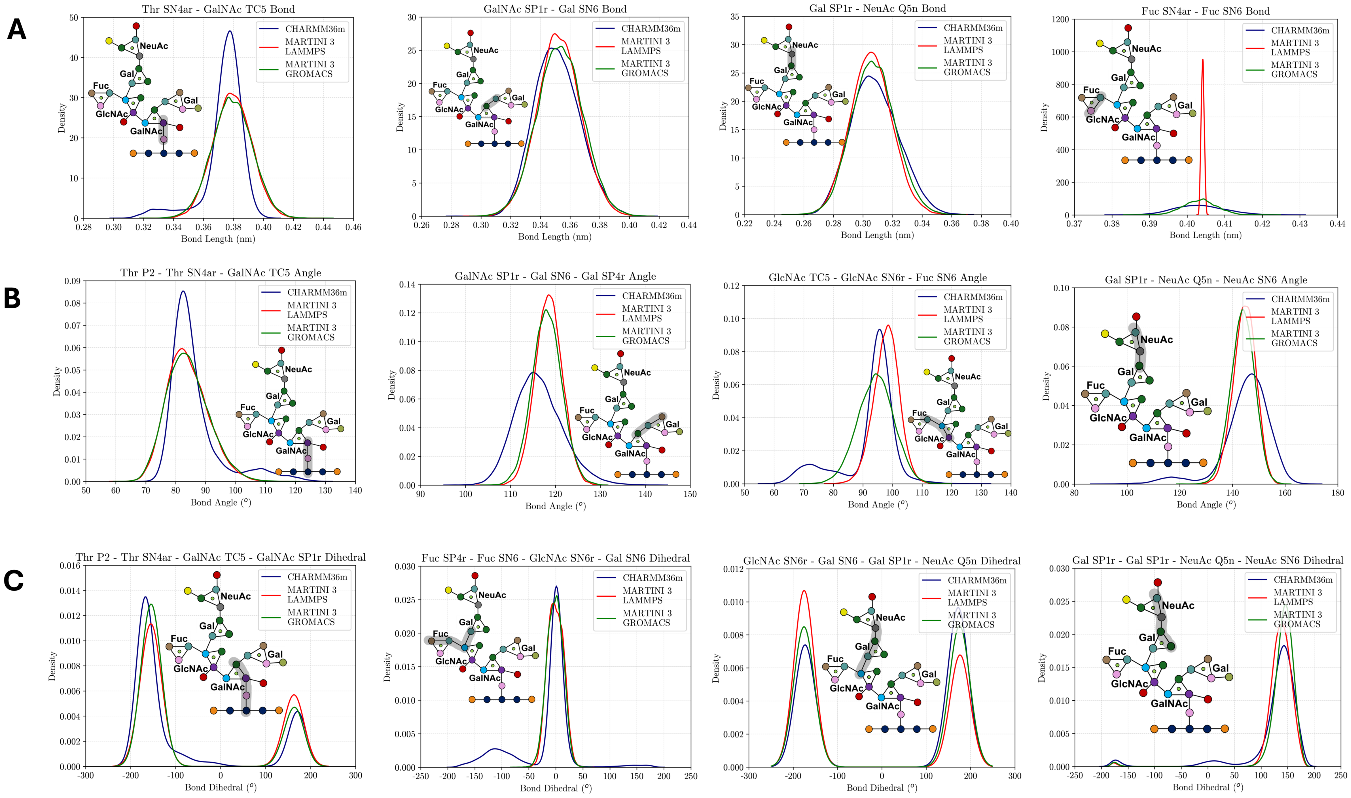

Figure S3: Distributions of selected bond lengths (A), angles (B) and dihedrals (C) within the G1 glycopeptide from atomistic MD simulations with CHARMM36m[3] (blue) and coarse-grained MD simulations with MARTINI 3[2] ran in LAMMPS[4] (red) and in GROMACS[5,6] (green). The depicted bonded term within each plot is highlighted in gray.

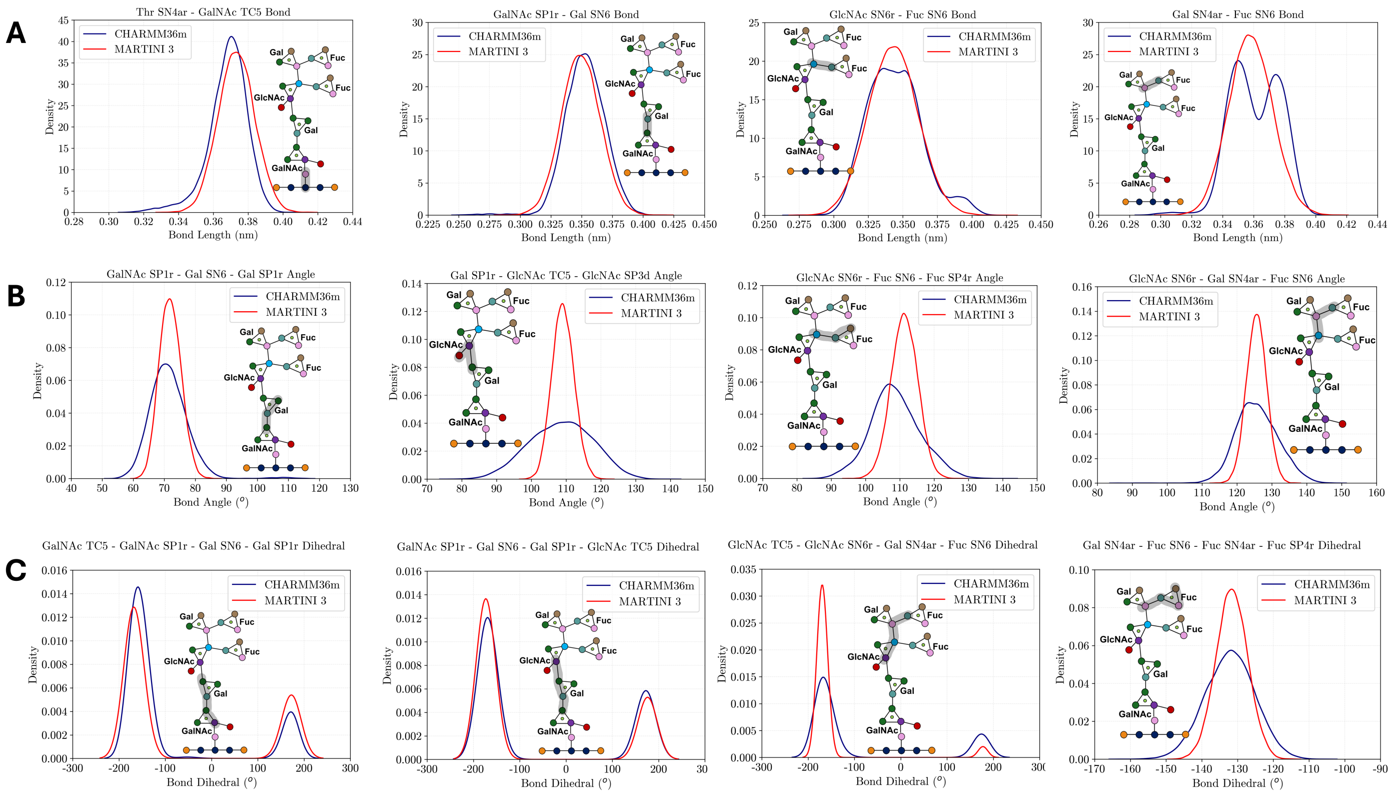

Figure S4: Distributions of selected bond lengths (A), angles (B) and dihedrals (C) within the G2 glycopeptide from atomistic MD simulations with CHARMM36m[3] (blue) and coarse-grained MD simulations with MARTINI 3[2] (red). The depicted bonded term within each plot is highlighted in gray.

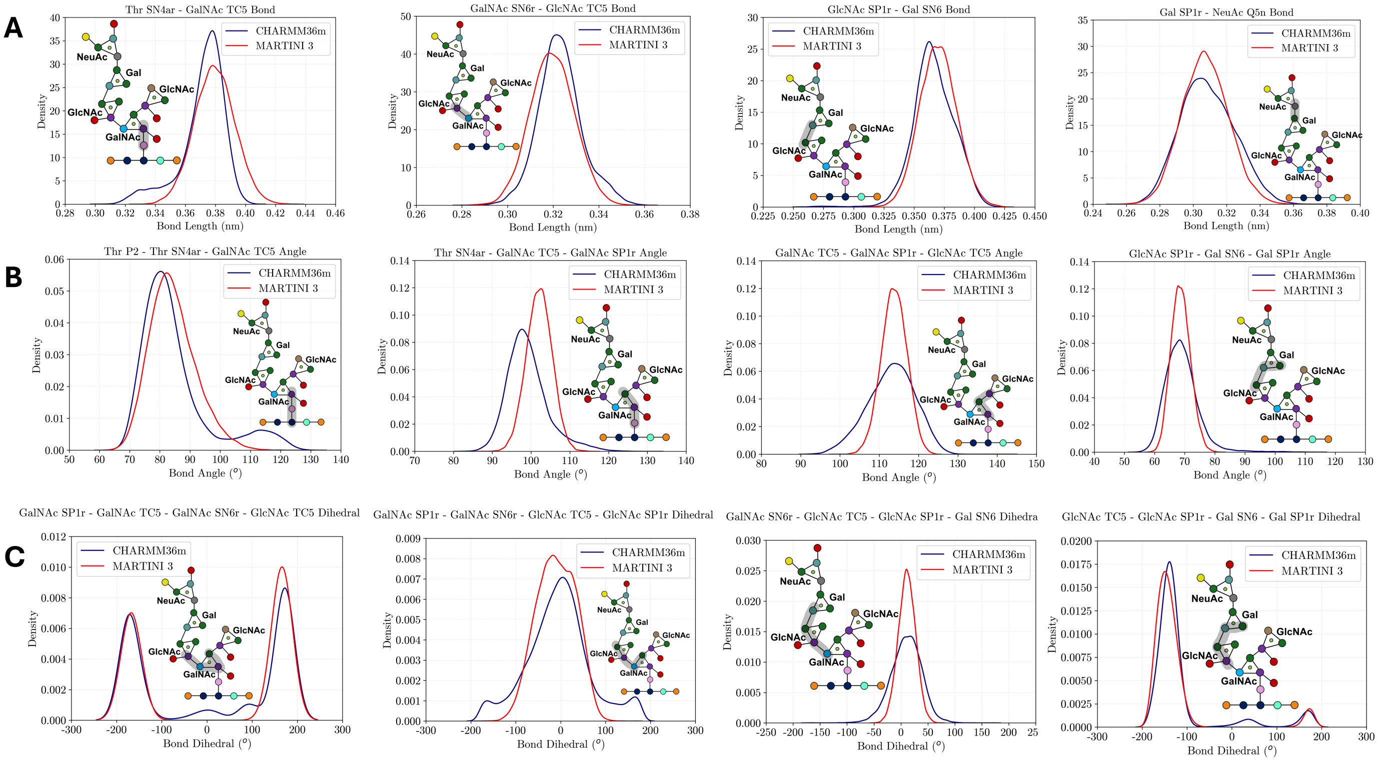

Figure S5: Distributions of selected bond lengths (A), angles (B) and dihedrals (C) within the G3 glycopeptide from atomistic MD simulations with CHARMM36m[3] (blue) and coarse-grained MD simulations with MARTINI 3[2] (red). The depicted bonded term within each plot is highlighted in gray.

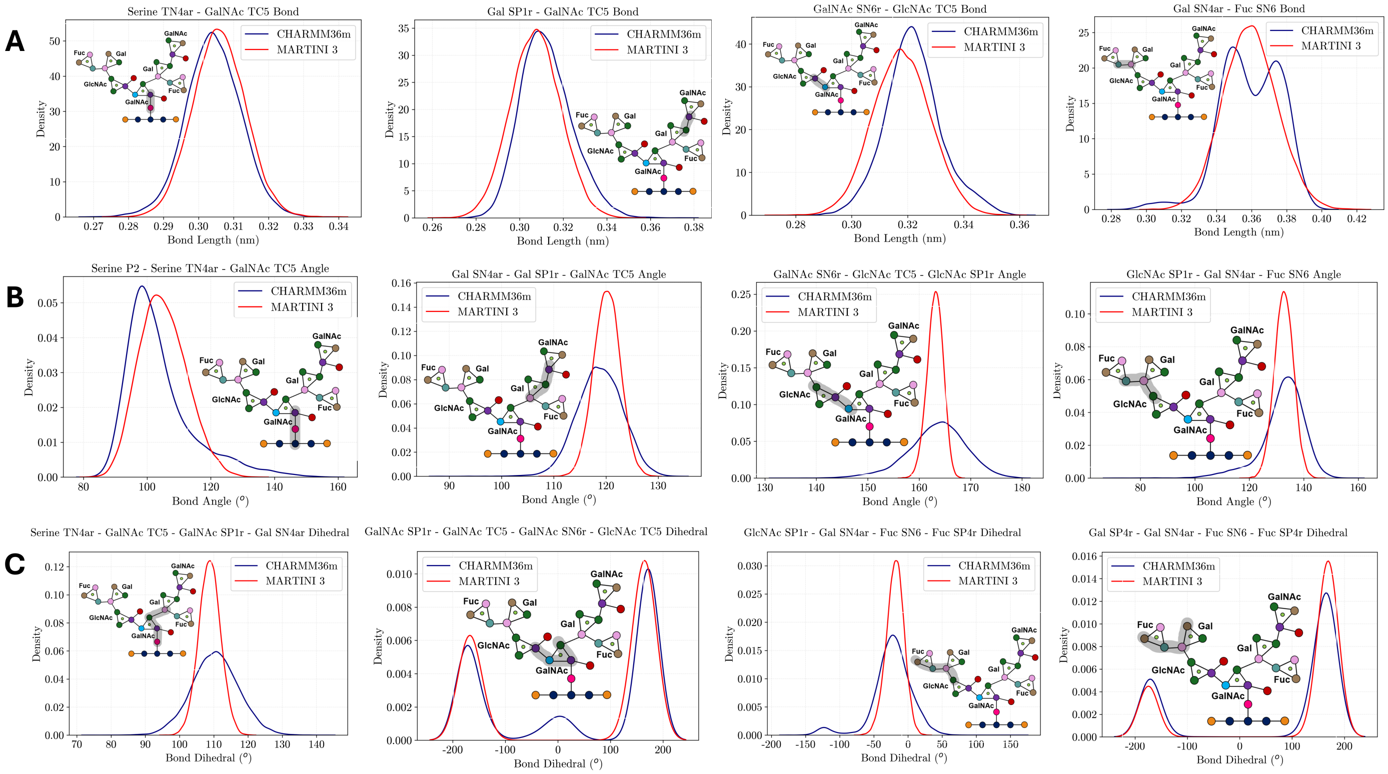

Figure S6: Distributions of selected bond lengths (A), angles (B) and dihedrals (C) within the G14 glycopeptide from atomistic MD simulations with CHARMM36m[3] (blue) and coarse-grained MD simulations with MARTINI 3[2] (red). The depicted bonded term within each plot is highlighted in gray.

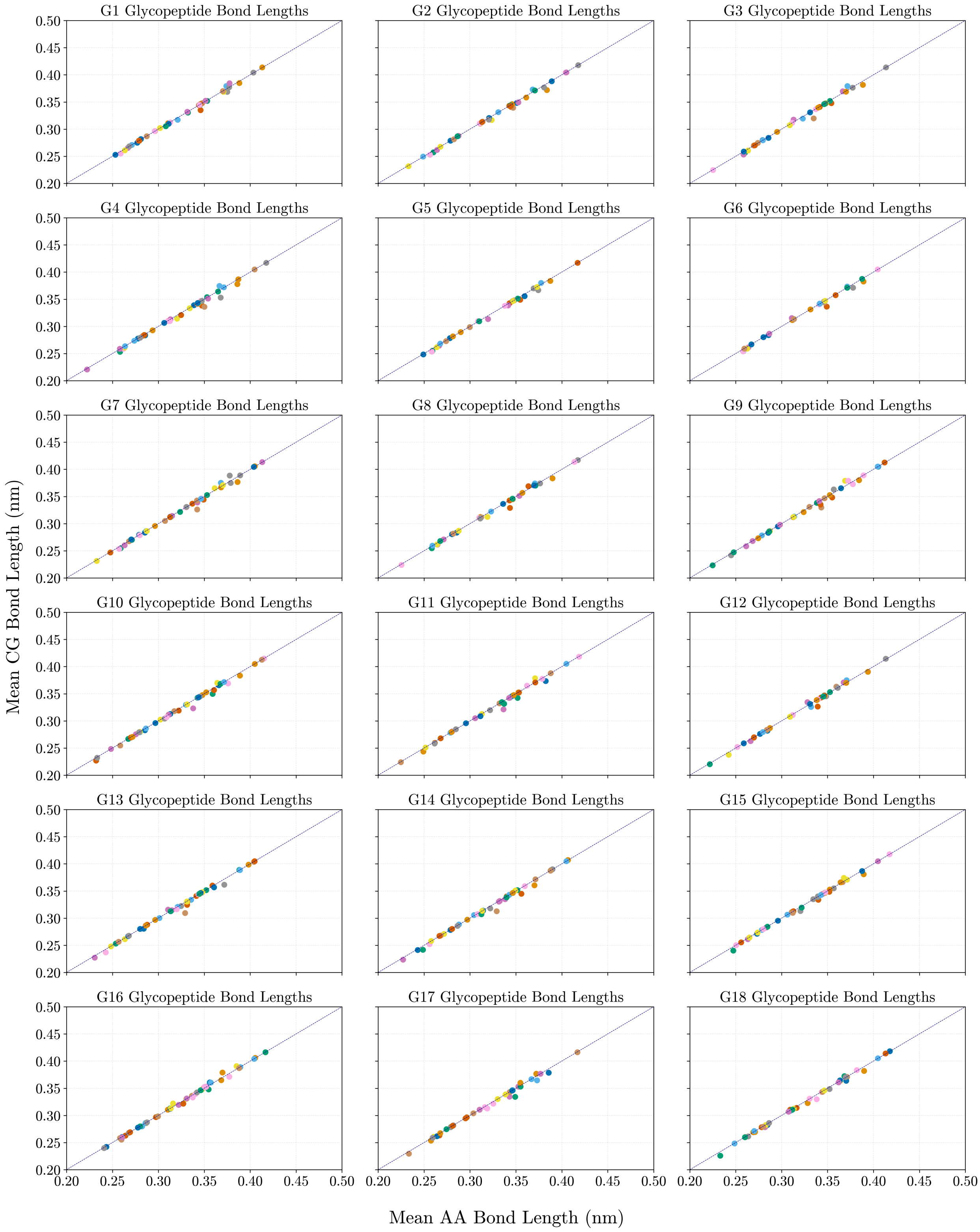

Figure S7: Parity plot comparing the mean bond lengths within each of the 18 glycopeptides between atomistic MD simulations with CHARMM36m[3] and coarse-grained simulations with MARTINI 3.[2]

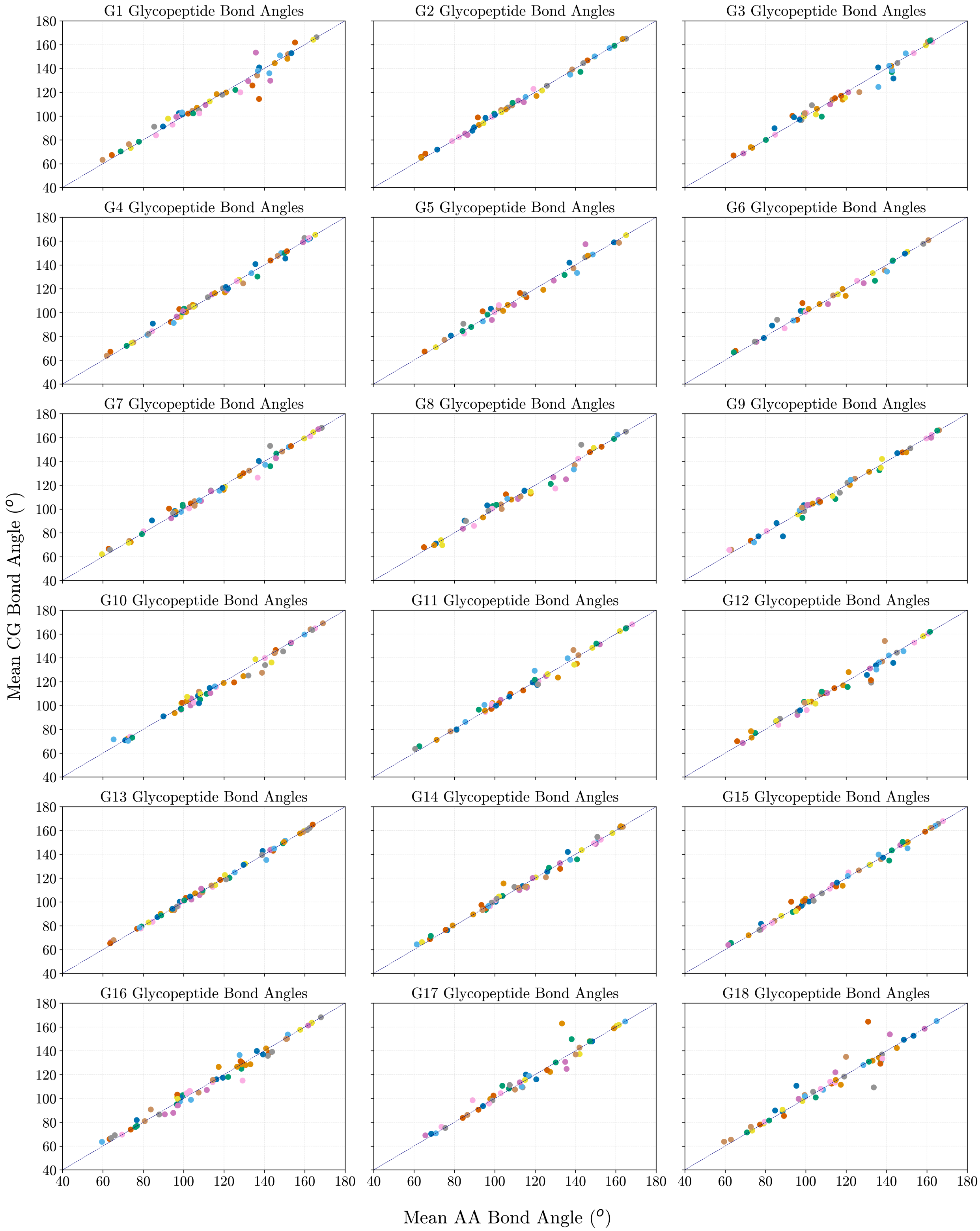

Figure S8: Parity plot comparing the mean bond angles within each of the 18 glycopeptides between atomistic MD simulations with CHARMM36m[3] and coarse-grained simulations with MARTINI 3.[2]

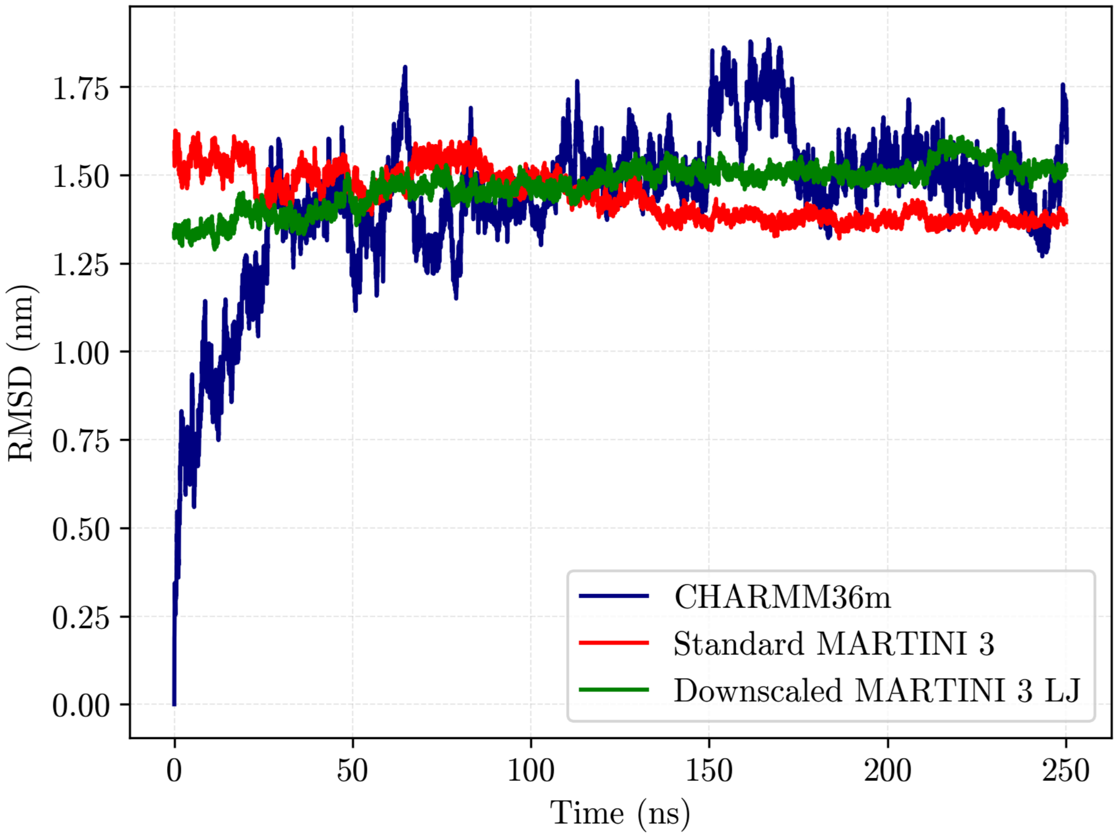

Figure S9: Time evolution of RMSD during the atomistic CHARMM36m simulation[3] (navy), the MARTINI 3 simulation with the standard LJ parameters[2] (red) and the MARTINI 3 simulation using downscaled LJ parameters[7] (green), using a pseudo-CG representation of the first frame of the atomistic trajectory as the reference.

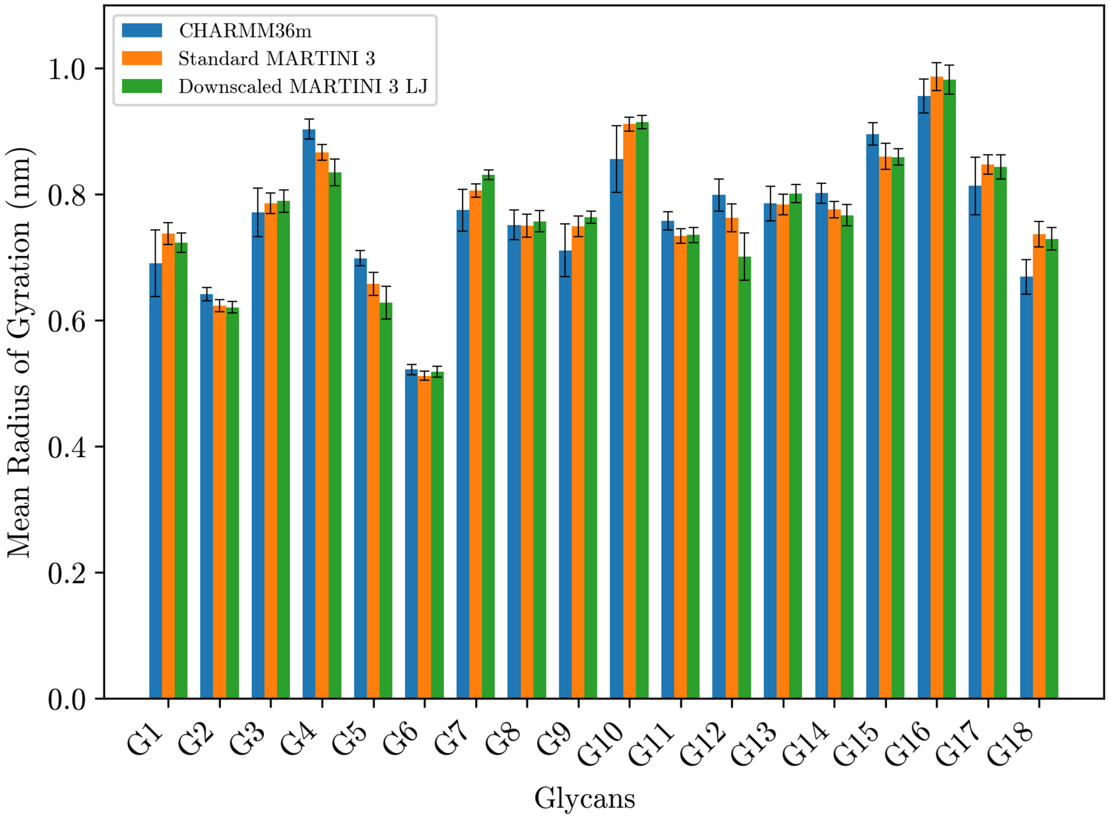

Figure S10: Mean Radius of Gyration of 18 glycans attached to the 30-amino acid MUC5B protein from the atomistic CHARMM36m simulation[3] (blue), the MARTINI 3 simulation with the standard LJ parameters[2] (orange) and the MARTINI 3 simulation using downscaled LJ parameters[7] (green).

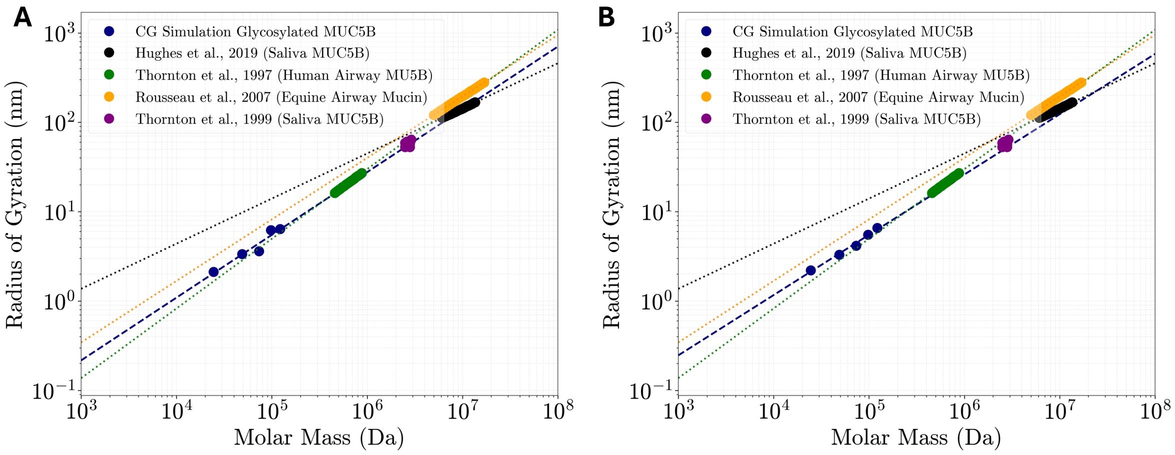

Figure S11: Scaling of the radius of gyration with molar mass for CG model of MUC5B run in GROMACS. Power-law fits to both the experimental data (dotted lines) and our simulation data (dashed lines) are illustrated with the scaling exponent, v, given below. Results are shown from simulations with the standard MARTINI 3 protein-protein LJ parameters[2] ($v = 0.702$) (A) and protein-protein LJ interactions downscaled by a factor of 0.88[7] ($v = 0.674$) (B). The results are compared with experimental data from Hughes et al.[8] (black, $v = 0.505$), Thornton et al.[9] (green, $v = 0.780$), Rousseau et al.[10] (orange, $v = 0.688$) and Thornton et al.[11] (purple).

References

(1) Kearns, F. L.; Rosenfeld, M. A.; Amaro, R. E. Breaking Down the Bottlebrush: Atomically Detailed Structural Dynamics of Mucins. *Journal of Chemical Information and Modeling* **2024**, *64*, 7949–7965.

(2) Souza, P. C. et al. Martini 3: a general purpose force field for coarse-grained molecular dynamics. *Nature Methods* **2021**, *18*, 382–388.

(3) Huang, J.; Rauscher, S.; Nawrocki, G.; Ran, T.; Feig, M.; Groot, B. L. D.; Grubmüller, H.; MacKerell, A. D. CHARMM36m: An improved force field for folded and intrinsically disordered proteins. *Nature Methods* **2016**, *14*, 71–73.

(4) Thompson, A. P.; Aktulga, H. M.; Berger, R.; Bolintineanu, D. S.; Brown, W. M.; Crozier, P. S.; in 't Veld, P. J.; Kohlmeyer, A.; Moore, S. G.; Nguyen, T. D.; Shan, R.;

Stevens, M. J.; Tranchida, J.; Trott, C.; Plimpton, S. J. LAMMPS - a flexible simulation tool for particle-based materials modeling at the atomic, meso, and continuum scales. *Computer Physics Communications* **2022**, *271*, 108171.

(5) Abraham, M. J.; Murtola, T.; Schulz, R.; Páll, S.; Smith, J. C.; Hess, B.; Lindah, E. GROMACS: High performance molecular simulations through multi-level parallelism from laptops to supercomputers. *SoftwareX* **2015**, *1-2*, 19–25.

(6) Van Der Spoel, D.; Lindahl, E.; Hess, B.; Groenhof, G.; Mark, A. E.; Berendsen, H. J. C. GROMACS: fast, flexible, and free. *Journal of Computational Chemistry* **2005**, *26*, 1701–1718.

(7) Thomasen, F. E.; Skaalum, T.; Kumar, A.; Srinivasan, S.; Vanni, S.; Lindorff-Larsen, K. Rescaling protein-protein interactions improves Martini 3 for flexible proteins in solution. *Nature Communications* **2024**, *15*, 6645.

(8) Hughes, G. W.; Ridley, C.; Collins, R.; Roseman, A.; Ford, R.; Thornton, D. J. The MUC5B mucin polymer is dominated by repeating structural motifs and its topology is regulated by calcium and pH. *Scientific Reports* **2019**, *9*, 17350.

(9) Thornton, D. J.; Howard, M.; Khan, N.; Sheehan, J. K. Identification of two glycoforms of the MUC5B mucin in human respiratory mucus. Evidence for a cysteine-rich sequence repeated within the molecule. *Journal of Biological Chemistry* **1997**, *272*, 9561–9566.

(10) Rousseau, K.; Kirkham, S.; Mckane, S.; Newton, R.; Clegg, P.; Thornton, D. J. Muc5b and Muc5ac are the major oligomeric mucins in equine airway mucus. *American Journal of Physiology-Lung Cellular and Molecular Physiology* **2007**, *292*, 1396–1404.

(11) Thornton, D. J.; Khan, N.; Mehrotra, R.; Howard, M.; Veerman, E.; Packer, N. H.; Sheehan, J. K. Salivary mucin MG1 is comprised almost entirely of different glycosylated forms of the MUC5B gene product. *Glycobiology* **1999**, *9*, 293–302.
